# Colonisation of hospital surfaces from low- and middle-income countries by extended spectrum β-lactamase- and carbapenemase-producing bacteria

**DOI:** 10.1038/s41467-024-46684-z

**Published:** 2024-03-29

**Authors:** Maria Nieto-Rosado, Kirsty Sands, Edward A. R. Portal, Kathryn M. Thomson, Maria J. Carvalho, Jordan Mathias, Rebecca Milton, Calie Dyer, Chinenye Akpulu, Ian Boostrom, Patrick Hogan, Habiba Saif, Ana D. Sanches Ferreira, Thomas Hender, Barbra Portal, Robert Andrews, W. John Watkins, Rabaab Zahra, Haider Shirazi, Adil Muhammad, Syed Najeeb Ullah, Muhammad Hilal Jan, Shermeen Akif, Kenneth C. Iregbu, Fatima Modibbo, Stella Uwaezuoke, Lamidi Audu, Chinago P. Edwin, Ashiru H. Yusuf, Adeola Adeleye, Aisha S. Mukkadas, Jean Baptiste Mazarati, Aniceth Rucogoza, Lucie Gaju, Shaheen Mehtar, Andrew N. H. Bulabula, Andrew Whitelaw, Lauren Roberts, Grace Chan, Delayehu Bekele, Semaria Solomon, Mahlet Abayneh, Gesit Metaferia, Group BARNARDS, Timothy R. Walsh

**Affiliations:** 1 Department of Biology, Ineos Oxford Institute for Antimicrobial Research, University of Oxford, Oxford, UK; 2 Division of Infection and Immunity, Cardiff University, Cardiff, UK; 3 Department of Medical Sciences, Institute of Biomedicine, University of Aveiro, Aveiro, Portugal; 4 Centre for Trials Research, Cardiff University, Cardiff, UK; 5 Parasites and Microbes Programme, Wellcome Sanger Institute Hinxton, Hinxton, UK; 6 Department of Microbiology, Quaid-i-Azam University, Islamabad, Pakistan; 7 Pakistan Institute of Medical Sciences, Islamabad, Pakistan; 8 National Hospital Abuja, Abuja, Nigeria; 9 Débbo Africa, Lekki, Lagos, Nigeria; 10 Federal Medical Centre Jabi, Abuja, Nigeria; 11 Department of Microbiology, Medway Maritime Hospital NHS Foundation Trust, Gillingham, Kent UK; 12 Aminu Kano Teaching Hospital, Kano, Nigeria; 13 Murtala Muhammad Specialist Hospital, Kano City, Nigeria; 14 The National Reference Laboratory, Rwanda Biomedical Centre, Kigali, Rwanda; 15 Unit of IPC, Stellenbosch University, Cape Town, South Africa; 16 Infection Control Africa Network, Cape Town, South Africa; 17 Department of Global Health, Stellenbosch University, Cape Town, South Africa; 18 Division of Medical Microbiology, Stellenbosch University, Cape Town, South Africa; 19 National Health Laboratory Service, Tygerberg Hospital, Cape Town, South Africa; 20 Department of Pediatrics, Boston Children’s Hospital, Harvard Medical School, Boston, MA USA; 21 Department of Pediatrics and Child Health, St Paul’s Hospital Millennium Medical College, Addis Ababa, Ethiopia; 22 Department of Epidemiology, Harvard T.H. Chan School of Public Health, Boston, USA; 23 Department of Obstetrics and Gynecology, St Paul’s Hospital Millennium Medical College, Addis Ababa, Ethiopia; 24 Department of Microbiology, Immunology and Parasitology, St Paul’s Hospital Millennium Medical College, Addis Ababa, Ethiopia

**Keywords:** Antibiotics, Bacterial genomics, Antimicrobial resistance

## Abstract

Hospital surfaces can harbour bacterial pathogens, which may disseminate and cause nosocomial infections, contributing towards mortality in low- and middle-income countries (LMICs). During the BARNARDS study, hospital surfaces from neonatal wards were sampled to assess the degree of environmental surface and patient care equipment colonisation by Gram-negative bacteria (GNB) carrying antibiotic resistance genes (ARGs). Here, we perform PCR screening for extended-spectrum β-lactamases (*bla*_CTX-M-15_) and carbapenemases (*bla*_NDM_, *bla*_OXA-48_-like and *bla*_KPC_), MALDI-TOF MS identification of GNB carrying ARGs, and further analysis by whole genome sequencing of bacterial isolates. We determine presence of consistently dominant clones and their relatedness to strains causing neonatal sepsis. Higher prevalence of carbapenemases is observed in Pakistan, Bangladesh, and Ethiopia, compared to other countries, and are mostly found in surfaces near the sink drain. *Klebsiella pneumoniae*, *Enterobacter hormaechei*, *Acinetobacter baumannii*, *Serratia marcescens* and *Leclercia adecarboxylata* are dominant; ST15 *K. pneumoniae* is identified from the same ward on multiple occasions suggesting clonal persistence within the same environment, and is found to be identical to isolates causing neonatal sepsis in Pakistan over similar time periods. Our data suggests persistence of dominant clones across multiple time points, highlighting the need for assessment of Infection Prevention and Control guidelines.

## Abstract (web summary)

In hospitals, surfaces present as a reservoir for bacteria pathogens, potentially leading to nosocomial infections. In this work, authors aim to profile extended-spectrum β lactamase- and carbapenemase-carrying bacterial species colonising neonatal hospital wards and causing neonatal sepsis.

## Introduction

Environmental surfaces and patient care equipment in hospital settings are among the most critical factors in bacterial horizontal transmission events from high-touch surfaces to patients^1–3^, with higher hospital-acquired infection (HAIs) rates in low-income and middle-income countries (LMICs) compared to high-income countries (HICs)^4^. Unless appropriate infection prevention and control (IPC) guidelines are applied and include effective cleaning, disinfection and hygiene practices^4–6^, environmental reservoirs of multidrug-resistant (MDR) bacteria can develop and direct contact with these may contribute to HAIs, such as surgical site infections and bloodstream infections^7–14^. High levels of antimicrobial resistance (AMR) can lead to difficulties in the treatment of MDR infections, associated with high mortality^15–18^. Neonates are particularly at risk because of their underdeveloped immune system, and neonatal infection rates in LMICs are 3–20 times higher than in HICs^19^. Furthermore, AMR bacteria with tolerance to disinfectants may survive on environmental surfaces for months, depending on factors such as humidity, temperature, air ventilation and surface characteristics^4,9,20^, which will further increase transmission likelihood.

Empirical treatment using cephalosporins or carbapenems in LMIC healthcare settings has influenced the progressive emergence of extended-spectrum β-lactamase- (ESBL-) and carbapenemase-producing bacteria. Antibiotic resistance genes (ARGs) such as *bla*_CTX-M-15_, and *bla*_NDM_, *bla*_OXA-48_-like or *bla*_KPC_ are often plasmid-borne and/or other mobile genetic element- (MGE) associated, enhancing potential for transmission^8^.

The most common ESBL- and carbapenemase-producing Gram-negative bacteria (GNB) in hospitals are *Enterobacterales*^1,3,8,11,12,21,22^. Few publications have shown data regarding the presence of GNB carrying β-lactamase genes on hospital surfaces in LMICs. Moreover, most of these studies performed in LMICs are single sites focused on specific countries, such as Bangladesh, Pakistan, Ethiopia, or Ghana^1,23–25^.

Here, we determine the prevalence and diversity of ESBL- and carbapenemase-carrying bacterial species colonising neonatal wards from six countries within the BARNARDS study (Burden of Antibiotic Resistance in Neonates from Developing Societies). Genetic lineages are assessed to determine whether transmission events occurred and whether they are related to bacteria causing neonatal sepsis in the BARNARDS study.

## Results

### GNB colonisation among countries and hospital sites

In total, 6290 hospital surface swabs (HSS) were processed from ten hospitals across six countries; and 60.7% HSS had GNB growth (Table 1). GNB colonisation varied significantly between the countries and hospital sites, with the highest growth in Bangladesh (92%).

**Table 1:** Gram-negative bacteria (GNB) colonisation per hospital sites and countries

Country/hospital	Samples with GNB colonisation	No growth	Total
Bangladesh	944 (92.19%)	80 (7.81%)	1024
BC	371 (90.49%)	39 (9.51%)	410
BK	573 (93.32%)	41 (6.68%)	614
Ethiopia (ES)	119 (47.04%)	134 (52.96%)	253
Nigeria	927 (59.2%)	639 (40.8%)	1566
NK	435 (92.16%)	37 (7.84%)	472
NN	273 (39.28%)	422 (60.72%)	695
NW	219 (54.89%)	180 (45.11%)	399
Pakistan (PP)	642 (62.15%)	391 (37.85%)	1033
Rwanda	538 (58.04%)	389 (41.96%)	927
RK	317 (67.88%)	150 (32.12%)	467
RU	221 (48.04%)	239 (51.96%)	460
South Africa (ZAT)	646 (43.44%)	841 (56.56%)	1487
Total	3816 (60.67%)	2474 (39.33%)	6290
Significance	*P* = 1.329^−134^		

Total number of samples collected per country and per hospital site were used as denominators to calculate the percentages. Additionally, a Chi-squared test was conducted to assess the extent of the overall differences in the proportions of GNB colonisation between the countries. GNB colonisation varied significantly (*P* < 0.001) between the six countries. *P* value of <0.05 was considered to show significant differences between the proportions. Abbreviations for BARNARDS hospitals detailed in the “Methods” section.

### ARG among countries and hospital sites

Overall, 839/6290 (13.3%) of HSS were positive for *bla*_CTX-M-15_, 338/6290 (5.4%) for *bla*_NDM_, and 74/6290 (1.2%) for *bla*_OXA-48_-like. *bla*_KPC_ was not detected during this study, therefore, from this point, the term ARGs denotes *bla*_CTX-M-15_*, bla*_NDM_ and *bla*_OXA-48_-like. Concomitant carriage of more than one ARG in the same sample was found in ~1% of samples (Supplementary Table 1).

**Fig. 1:**
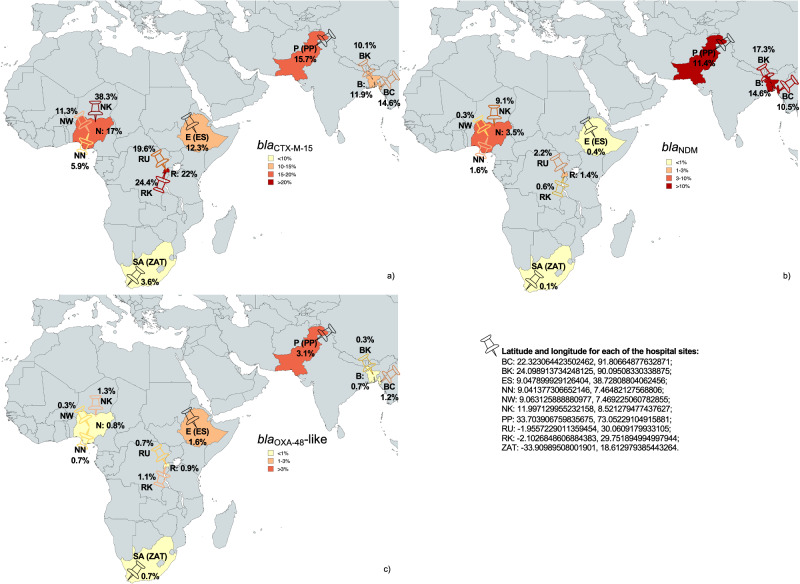
Maps indicating prevalence of ARG per site and country. Prevalence of *bla*_CTX-M-15_ (**a**), *bla*_NDM_ (**b**), and *bla*_OXA-48_-like (**c**) genes per country and per hospital site. The total number of samples collected per country and per hospital site were used as denominators to calculate the prevalence of each antibiotic resistance gene (ARG) per country and per hospital site, respectively. Abbreviations for BARNARDS hospitals are detailed in the “Methods” section, map pins showing latitude and longitude, and coloured according to the ARG prevalence in that site. Source data are provided in Supplementary Table 2.

The prevalence of *bla*_CTX-M-15_, *bla*_NDM_ and *bla*_OXA-48_-like ranged between countries (Fig. 1 and Supplementary Table 2). Noticeable differences between hospitals were observed; for instance, in Bangladesh, almost twice as many *bla*_NDM_ were detected in BK (17.3%) compared to BC (10.5%), whereas *bla*_OXA-48_-like was four times more frequent in BC (1.2%). Hospital surfaces in RU had higher *bla*_NDM_ (2.2%) rates compared to RK, and conversely, *bla*_CTX-M-15_ and *bla*_OXA-48_-like were more prevalent (24.4% and 1.1%, respectively) in RK. In Nigeria, the prevalence of all the ARGs was highest in one site (NK).

### GNB colonisation and ARG detection among hospital surfaces

To study the correlation between the presence of ARG and surface type, *n* = 4126/6290 samples contained appropriate metadata following data cleaning (Table 2). The 4126 HSS were collected from 309 different surfaces (Supplementary Data 1) and classified into six categories (listed in Table 2; see methods section “Data cleaning and statistical analysis” for details).

**Table 2:** Number of hospital surface swabs (HSS) collected per country and per hospital site, over the total of 4126 HSS (where information regarding surface was available)

Country	Hospital site	Patient’s zone	Surfaces near the sink drain	Emergency neonatal care	Ward furniture/surfaces	Mobile medical equipment	Medical equipment	Total
Bangladesh	BC	1	73	11	73	78	19	255
	BK	82	26	0	78	57	1	244
	Total B	83	99	11	151	135	20	499
Ethiopia	ES	41	54	32	80	16	22	245
	Total E	41	54	32	80	16	22	245
Nigeria	NK	28	5	15	19	11	34	112
	NN	139	67	133	83	90	146	658
	NW	202	12	89	47	10	16	376
	Total N	369	84	237	149	111	196	1146
Pakistan	PP	160	42	121	76	135	247	781
	Total P	160	42	121	76	135	247	781
Rwanda	RK	50	38	179	69	50	15	401
	RU	35	37	131	79	58	54	394
	Total R	85	75	310	148	108	69	795
South Africa	ZAT	87	105	22	152	42	252	660
	Total SA	87	105	22	152	42	252	660
TOTAL		825	459	733	756	547	806	4,126

Abbreviations for BARNARDS hospitals are detailed in the “Methods” section.

Table 3a summarises the differences in GNB growth across surface categories observing the largest growth near sink drains (68.4%). HSS collected from medical equipment were the least contaminated with Gram-negative bacterial colonisation (45.9%). Furthermore, HSS positive for *bla*_CTX-M-15_ and *bla*_OXA-48_-like varied significantly (*P* < 0.001 and *P* = 0.004, respectively) between the six surface categories (Table 3b). Although the presence of *bla*_NDM_ varied, this was not statistically significant (*P* = 0.071), and the ARG presence was higher in surfaces classified as near the sink drain (Table 3). For detailed ARG prevalence on the different 309 surface types collected and on surface categories among all hospital sites, see Supplementary Data 2 and 3, respectively.

**Table 3:** Statistical analysis to identify correlation between hospital surfaces and Gram-negative bacteria (GNB) colonisation and antibiotic resistance genes (ARGs)

(a)	GNB colonisation		
	Positive	Negative	Total
Patient’s zone	511 (61.94%)	314 (38.06%)	825
Surfaces near the sink drain	314 (68.41%)	145 (31.59%)	459
Emergency neonatal care	379 (51.71%)	354 (48.29%)	733
Ward furniture/surfaces	474 (62.7%)	282 (37.3%)	756
Mobile medical equipment	343 (62.71%)	204 (37.29%)	547
Medical equipment	370 (45.91%)	436 (54.09%)	806
Total	2391 (57.95%)	1735 (42.05%)	4126

(b)		*bla*_CTX-M-15_		*bla*_NDM_		*bla*_OXA-48_-like		
		Negative	Positive	Negative	Positive	Negative	Positive	Total
Patient’s zone	Count	702	123	786	39	815	10	825
	Row *N* %	85.1%	14.9%	95.3%	4.7%	98.8%	1.2%	100.0%
Surfaces near the sink drain	Count	365	94	425	34	444	15	459
	Row *N* %	79.5%	20.5%	92.6%	7.4%	96.7%	3.3%	100.0%
Emergency neonatal care	Count	607	126	705	28	724	9	733
	Row *N* %	82.8%	17.2%	96.2%	3.8%	98.8%	1.2%	100.0%
Ward furniture /surfaces	Count	652	104	725	31	750	6	756
	Row *N* %	86.2%	13.8%	95.9%	4.1%	99.2%	0.8%	100.0%
Mobile medical equipment	Count	476	71	521	26	543	4	547
	Row *N* %	87.0%	13.0%	95.2%	4.8%	99.3%	0.7%	100.0%
Medical equipment	Count	723	83	773	33	797	9	806
	Row *N* %	89.7%	10.3%	95.9%	4.1%	98.9%	1.1%	100.0%
**Total**	Count	3525	601	3935	191	4073	53	4126
	Row *N* %	85.4%	14.6%	95.4%	4.6%	98.7%	1.3%	100.0%
Significance		*P* = 0.000013		*P* = 0.071		*P* = 0.004		

Chi-squared test was conducted to assess the extent of the differences in the proportions of ARGs between the surface categories. (a) Frequency of positive and negative GNB growth among hospital surfaces. (b) Contingency table showing the percentage of negative and positive samples for ARG among hospital surfaces. Significant differences (*P* < 0.05) were observed when comparing the prevalence of each ARG across surfaces; *bla*_CTX-M-15_ and *bla*_OXA-48_-like frequency varied significantly between the six surface categories (*P* < 0.05), but no significant differences in presence across surfaces were observed for *bla*_NDM_ (*P* > 0.05).

To study the correlation between the presence of ARG and a particular time of the year, *n* = 4662/6290 samples contained appropriate metadata following data cleaning. Temporal analysis revealed that the percentage of *bla*_CTX-M-15_, *bla*_NDM_ and *bla*_OXA-48_-like varied significantly between the seven-time bands across the sampling period from November 2015 until January 2018 (*P* < 0.001 for *bla*_CTX-M-15_ and *bla*_NDM_, and *P* = 0.041 for *bla*_OXA-48_-like) (Table 4). GNB HSS colonisation appeared to be highest during the March–June and July–October periods.

**Table 4:** Distribution of antibiotic resistance genes (ARGs) prevalence and Gram-negative bacteria (GNB) colonisation according to the timeline (November 2015–January 2018)

Timeline	*bla*_CTX-M-15_	*bla*_NDM_	*bla*_OXA-48_-like	GNB colonisation	Total HSS collected in each time band
Nov15–Feb16	32 (10.36%)	16 (5.18%)	2 (0.65%)	159 (51.46%)	309
Mar16–Jun16	110 (15.87%)	42 (6.06%)	12 (1.73%)	456 (65.8%)	693
Jul16–Oct16	94 (11.49%)	30 (3.67%)	6 (0.73%)	459 (56.11%)	818
Nov16–Feb17	61 (6.02%)	57 (5.62%)	10 (0.99%)	534 (52.66%)	1014
Mar17–Jun17	126 (18.31%)	84 (12.21%)	16 (2.33%)	501 (72.82%)	688
Jul17–Oct17	188 (21.05%)	43 (4.82%)	7 (0.78%)	623 (69.76%)	893
Nov17–Jan18	28 (11.34%)	16 (6.48%)	4 (1.62%)	141 (57.09%)	247

Chi-squared test was conducted to assess the extent of the differences in the proportions of ARGs between the time bands. The total of samples collected during each time band were used as denominators to calculate the percentages. The percentage of the ARGs varied significantly between the time bands (*P* *=* 2.89^−22^ for *bla*_CTX-M-15_, *P* *=* 2.82^−10^ for *bla*_NDM_, and *P* *=* 0.041 for *bla*_OXA-48_-like). GNB colonisation also varied significantly (*P* = 4.98^−25^). *P* value was considered statistically significant when *P* < 0.05.

### GNB species carrying *bla*_NDM_ and *bla*_OXA-48_-like

**Fig. 2:**
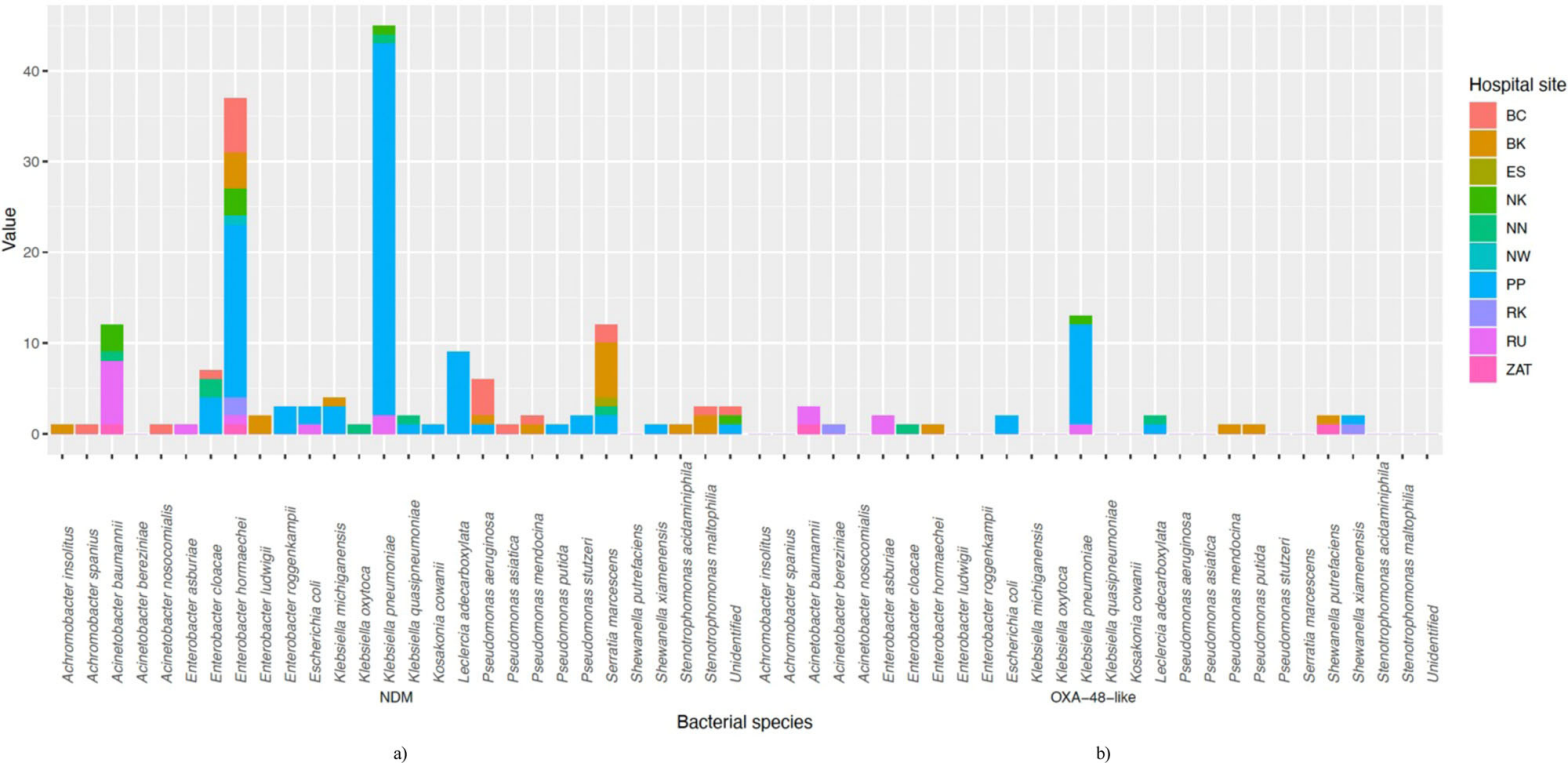
Bacterial species diversity carrying carbapenemase genes. Bacterial species carrying *bla*_NDM_ (**a**) and *bla*_OXA-48_-like (**b**) among countries and sites, according to PCR screening and MALDI-TOF MS identification (a total of 175 bacterial isolates from 27 bacterial species; and 3 “unidentified” isolates). Source data are provided in Supplementary Data 4.

In total, 175 bacterial isolates from 151/6,290 HSS with a positive multiplex-PCR for a carbapenemase ARG were purified. MALDI-TOF MS identified 27 different bacterial species, with *Klebsiella* spp. and *Enterobacter* spp. carrying *bla*_NDM_ and/or *bla*_OXA-48_-like equally dominant (*n* = 53/175, 30.3%). Within the *Klebsiella* spp., *K. pneumoniae* was most prevalent, accounting for 86.8% (*n* = 46/53). Similarly, within the *Enterobacter* spp. isolates recovered, *E. hormaechei* was the most frequent (*n* = 38/53, 71.7%), followed by *E. cloacae* (*n* = 8/53, 15.1%). Other GNBs identified include *Acinetobacter baumannii* (*n* = 13/175, 7.4%), *Pseudomonas* spp. (*n* = 14/175, 8%), *Serratia marcescens* (*n* = 12/175, 6.9%) and *Leclercia adecarboxylata* (*n* = 11/175, 6.3%) (Fig. 2). Within the dataset, only three *E. coli* isolates carrying carbapenemase ARG were recovered (*n* = 3/175, 1.7%, Supplementary Data 4 and Supplementary Table 3).

*bla*_NDM_-producing *K. pneumoniae* and *E. hormaechei* isolates were mostly found in Pakistan and altogether represented over 60% of the 93 carbapenemase-producing isolates found in PP (Supplementary Data 4). Supplementary Table 3 shows detailed information on surfaces where these isolates were recovered from.

### Bacterial diversity and whole genome sequencing (WGS) analysis of ARG variants

Whole genome sequencing (WGS) data was available for 128 carbapenemase-positive GNB isolates out of the 175 total isolates recovered from HSS (due to loss of growth or loss of carbapenemase gene upon regrowth), and of these, 18 bacterial species were identified (Supplementary Data 5).

**Fig. 3:**
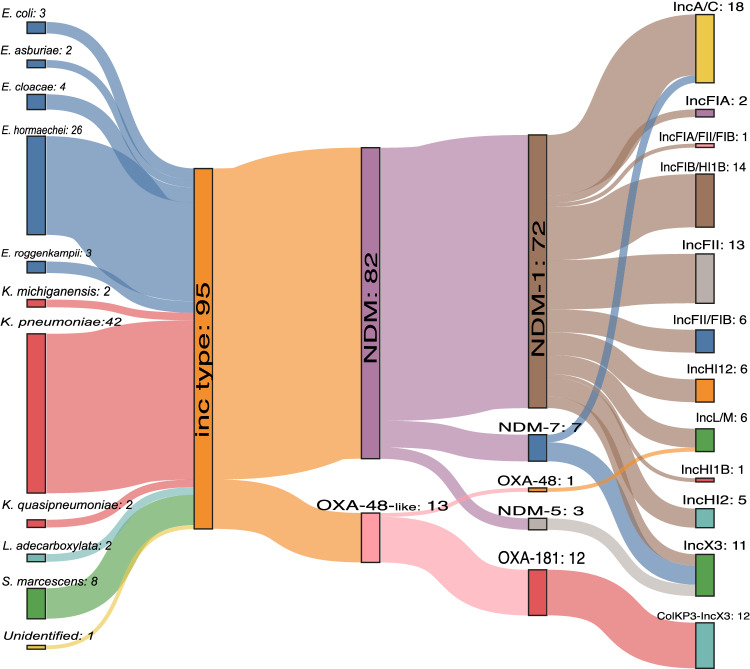
A Sankey diagram linking bacterial species, plasmids and carbapenemase ARGs. There were *n* = 95 isolates with an identifiable Inc plasmid type (due to assembly fragmentation, it was not always possible to assemble and analyse whole plasmids). The carbapenemase variant NDM and OXA-48-like group are divided into variants, and the Inc type detected per ARG-variant is shown. Source data are provided in Supplementary Data 5 and 6 and as Source Data file.

Of the GNB isolates with WGS data, 122 carried at least one *bla*_NDM_ variant (*n* = 114 *bla*_NDM-1_, n = 3 *bla*_NDM-5_, and *n* = 7 *bla*_NDM-7_ genes) and 17 isolates carried *bla*_OXA-48-_like genes (*n* = 14 *bla*_OXA-181_, *n* = 2 *bla*_OXA-204_, *n* = 1 *bla*_OXA-48_). The majority of carbapenemase ARGs were found to be plasmid-mediated (Fig. 3), however, for 16 isolates, the ARGs were chromosomally located (Supplementary Data 6).

Twelve isolates concomitantly carried variants of *bla*_NDM_ and *bla*_OXA-48_-like. This was most notably detected within sequence type (ST) 15 *K. pneumoniae* (*n* = 10/16 from Pakistan), with *n* = 9/10 carrying both *bla*_NDM-1_ and *bla*_OXA-181_ on similar ~140 kbp *Inc*A/C and ~50 kpb ColKP3-IncX3 hybrid plasmids respectively (Fig. 3, Supplementary Figs. 1–3); *n* = 1/10 carried both variants, but the plasmid type remained unknown due to assembly fragmentation.

Two ST11 *K. pneumoniae* isolates from Pakistan carried two *bla*_NDM_ genes: one harboured two copies of *bla*_NDM-7_ on a 30 kbp *Inc*X3 and 145 kbp *Inc*A/C plasmids, and the other one carried *bla*_NDM-1_ (on a ~39 kpb *Inc*X3 plasmid) and *bla*_NDM-7_ on a ~145 kpb *Inc*A/C plasmid (Fig. 3).

The other two *Klebsiella* species that were identified were *K. quasipneumoniae* and *K. michiganensis*. Plasmids represented in Supplementary Figs. 1, 2 from were from multiple bacterial species, including *K. pneumoniae, K. michiganensis* and *Enterobacter roggenkampii*.

Of the six isolates with *bla*_NDM-7_, three were *K. pneumoniae* and three were *E. hormaechei*.

From the 47 *Enterobacter* isolates, *E. hormaechei* was dominant (*n* = 38). Supplementary Data 6 shows the distinct ST recovered, with 21 STs for *Enterobacter* alone, with the most frequent being ST120, ST231, ST316 and ST418, all *E. hormaechei*. Three *E. hormaechei* isolates with different STs carrying a *bla*_NDM-5_ on *Inc*X3 plasmids (ranging from 46 to 55 kb; Fig. 3) were recovered from a single hospital (NK) in the same month in 2016 (Supplementary Data 5 shows the recovery date of the isolates with WGS data available). The two 46 kb plasmids (pNK-E166-IncX3 and pNK-E171A-IncX) shared 99.7% sequence homology. Supplementary Figs. 3, 4 show also that the 55 kb pNK-E179A-IncX3 was more genetically distant (30–31% aligned homologous sequences). Eight *Enterobacter* spp. isolates, mostly ST316 *E. hormaechei* (*n* = 5) co-carried *bla*_NDM-1_ and *mcr-9* on a large 400kbp *Inc*HI2 plasmid. *S. marcescens* was most often recovered in both BC and BK, all *n* = 9 isolates carried *bla*_NDM-1_, either on *Inc*F or *Inc*L/M plasmid types (Fig. 3).

One ST78 *A. baumannii* with two copies of *bla*_NDM-1_ and four ST52 *A. baumannii* isolates were recovered from RU and the hospital in South Africa (ZAT). Of the two *E. coli* isolates (ST448 and ST405) identified by WGS, both carried *bla*_NDM-1_ and one (ST405) co-carried *bla*_NDM-1_ and *bla*_OXA-181_.

WGS confirmed the presence of multiple aminoglycosides (*aac, ant, aph*), ESBLs (*bla*_OXA-1_, *bla*_SHV-11,_ *bla*_SHV-12,_ *bla*_SHV-106,_ *bla*_SHV-182_ and *bla*_SHV-187_), fosfomycin (*fosA)* and tetracycline (*tetA, tetB, tetD*) ARGs. All isolates were additionally screened for genes conferring resistance to disinfectants, and *MexAB-OprM, MexCD-OprJ, MexEF-OprN* and *MexJK-OpmH* were identified in three *Pseudomonas* spp. isolates, two from Bangladesh and one from Pakistan.

### Evidence of local transmission and links to neonatal sepsis

**Fig. 4:**
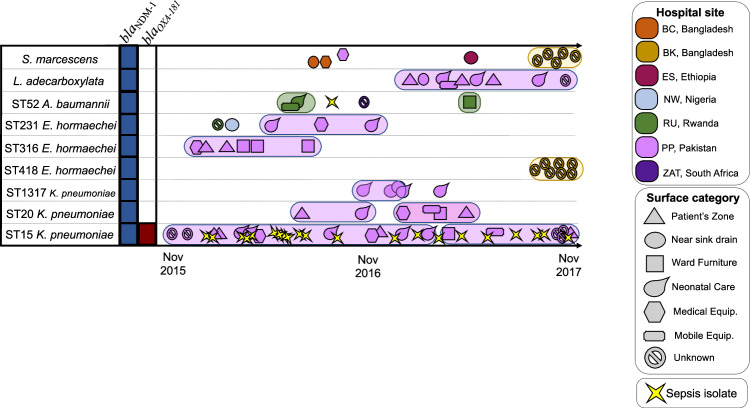
Timeline showing nine potential clusters of bacterial isolates recovered from hospital surface swabs (HSS) and whether the same strain of bacteria was found in sepsis isolates during BARNARDS. Sequence types (STs) and antimicrobial resistance genes (ARGs) are shown, and the highlighted background around isolates indicated these were within 10 pairwise SNPs. The different pink tone for ST20 *K. pneumoniae* indicates distinct sub-clusters identified among these isolates in Pakistan (over 1000 SNPs). Abbreviations for BARNARDS hospitals are detailed in the “Methods” section. The figure was created using Adobe Illustrator v26.5. Source data are provided in Supplementary Data 5 and 6.

From WGS and epidemiology analysis, nine potential cluster/transmission events were analysed by SNP analysis (Fig. 4). HSS isolates from two clusters shared the same ST of isolates from the same hospital site causing neonatal sepsis during BARNARDS (ST15 *K. pneumoniae* and ST52 *A. baumannii* isolates). WGS data from these sepsis isolates were included in the single nucleotide polymorphism analysis (total of *n* = 22 ST 15 *K. pneumoniae* isolates causing sepsis in Pakistan)^26^.

For ST15 *K. pneumoniae*, there were 16 HSS isolates analysed alongside 22 ST15 *K. pneumoniae* isolates from neonatal blood cultures within PP during the same time-period (November 2015–November 2017). ST15 *K. pneumoniae* were recovered from HSS from emergency neonatal care (*n* = 5), patient’s zone (*n* = 4), ward furniture/surfaces (*n* = 1), medical equipment (*n* = 1), and NA (*n* = 5). SNP analysis revealed that, except for one isolate, ST15 *K. pneumoniae* from Pakistan from both HSS and neonatal sepsis blood cultures (*n* = 22) were within 10 pairwise SNPs. Interestingly, in eight HSS isolates, *bla*_OXA-181_ and the ColKP3–*Inc*X3 hybrid replicon were not detected, indicating that some isolates might not have acquired the ColKP3-*Inc*X3 plasmid or may have lost the plasmid during culture for WGS. Furthermore, the ST405 *E. coli* isolates from the same hospital wards in Pakistan carried genetically similar plasmids, both the *Inc*A/C *bla*_NDM-1_ and the ColKP3-*Inc*X3 *bla*_OXA-181_ plasmids suggesting plasmid transmission may have occurred between bacterial species colonising hospital surfaces. It is possible that the ST405 *E. coli* isolate acquired both *bla*_NDM-1_ and *bla*_OXA-181_ from ST15 *K. pneumoniae*, as our data indicates multiple hospital surfaces were contaminated with this strain between 2016 and 2017 (Supplementary Figs. 5–8).

From nine *A. baumannii* isolates with WGS data, five were ST52, and SNP analysis of ST52 revealed the sepsis isolate (identified in the same hospital in Rwanda (RU)^26^) to be genetically distant (>100 SNPs) from the isolates collected from HSS, which were within 10 SNPs.

Isolates of ST20 and ST1317 *bla*_NDM-1_ *K. pneumoniae* were within 10 pairwise SNPs among isolates from each cluster, further evidencing the colonisation and potential transmission across the hospital wards in Pakistan. For ST20 isolates, there were two small sub-clusters identified, with genetic grouping occurring for isolates collected in late 2016 (September–November) and a distinct cluster of over 1000 SNPs distinct for ST20 *K. pneumoniae* recovered between January and March 2017 (Fig. 4).

*E. hormaechei*, the dominant *Enterobacter* species, was mostly identified in Asia. Clusters of ST231 and ST316 (*bla*_NDM-1_ and *mcr-9*) *E. hormaechei* isolates recovered across surfaces in PP were within three and two pairwise SNPs. A cluster of seven ST418 *E. hormaechei* HSS isolates from BK, recovered during a 6-week period between October and November 2017, were within four pairwise SNPs.

A cluster of five BK *S. marcescens* isolates (within 3 SNPs) was over 10,000 SNPs distant from the *S. marcescens* isolates from NN and BC. Nine *L. adecarboxylata* isolates isolated between March and November 2017 from different surfaces in PP were closely related (within a single SNP distance) (Fig. 4).

## Discussion

Herein, we report a high prevalence of bacteria carrying *bla*_CTX-M-15_, *bla*_NDM_ and *bla*_OXA-48_-like genes colonising environmental surfaces and patient care equipment in 10 hospitals across six LMICs. To the best of our knowledge, this multinational study is the first to evidence transmission networks between hospital wards and neonates with sepsis in LMICs^26^. Importantly, a high-risk double carbapenemase ST15 *K. pneumoniae* clone^8,27^ was recovered from HSS and blood cultures from septic neonates in Pakistan over a 2-year period, suggesting this strain was colonising surfaces in the ward whilst simultaneously causing neonatal sepsis^26^. In our dataset, we identified a single *E. coli* isolate carrying a genetically similar *bla*_NDM_ (*Inc*AC plasmid type) plasmid to the ST15 *K. pneumoniae* strain isolated from the same hospital (PP). We evidence similarity between plasmids detected within *Enterobacterales* from multiple hospital surfaces sampled between May 2016 and November 2017, indicating possible horizontal transmission however, a larger genomics dataset would be needed to understand plasmid transmission dynamics upon hospital surface samples (e.g. plasmids harboured in *E. hormaechei* isolates from NK).

ST15 *K. pneumoniae* has been reported to be responsible for neonatal sepsis and high mortality in clinical settings^8^, is often linked with HAIs and hospital outbreaks^28–30^ and is frequently detected in the environment (wastewater and soil) in Africa and Asia^31,32^ Furthermore, multiple clusters of the same strain of different GNB species were detected from hospital surfaces in Bangladesh and Rwanda, suggesting that hospital surface colonisation by pathogenic bacteria is a significant concern in LMICs hospitals. WHO and Médecins Sans Frontières still lack AMR data from many LMIC settings, thus, global reports on IPC, burden of sepsis or HAIs contain information mostly generated from HICs. This data highlights the widespread bacterial colonisation and the transmission of bacteria carrying multiple ARG upon hospital surfaces, which could be useful to guide realistic approaches and support action plans for countries where IPC practices are limited^13,18,19,22,33,34^.

Previous studies report that frequently used medical equipment/touch surfaces in healthcare settings are crucial for the cross-transmission of AMR bacteria and HAIs, such as sepsis^3,7,17,21,22,25,35^. This is particularly true in institutions where resources to implement IPC programs are scarce^4,13^. Bacteria carrying ARGs appeared to be found most frequently on surfaces near the sink drain, which was concordant with the work by Firesbhat et al. in Ethiopia^16^. We also found contamination of medical equipment and/or ward furniture/surfaces, as detected in other African-based studies reporting on HAIs^24,36–38^.

Interestingly, ESBL- and carbapenemase-producing *Enterobacterales* were more frequently detected in HSS collected between March and October. Despite the potential contamination pattern during this period, due to inconsistent sampling throughout the hospital sites among time periods, detailed seasonal analysis per country could not be performed herein. Apart from unequal sample size, a variety of factors, including temperature, cleaning practices, or healthcare workers shifts, may be influencing bacterial reservoirs at particular times of the year.

Our findings are comparable with previous studies in Pakistan^25^, Nigeria^36^ and Ethiopia^24,39^, which report >60% bacterial growth on medical equipment and environmental surfaces. We observed large differences in GNB growth between countries or hospitals within the same country, emphasising that bacterial colonisation should be monitored in each hospital. Apart from the sampling limitations of this study, there are likely various factors contributing to the range of colonisation observed, including antibiotic accessibility^40^.

In this study, *K. pneumoniae*, *E. hormaechei*, *A. baumannii*, *S. marcescens* and *L. adecarboxylata* were the most prevalent bacterial species carrying *bla*_CTX-M-15_, *bla*_NDM_ and *bla*_OXA-48_-like genes across all countries, which have been commonly described^1,3,8,11,17,24,25,35,39^. WGS revealed diversity within species showing multiple and co-occurring dominant STs of *E. hormaechei* and *K. pneumoniae*. MDR *A. baumannii* carrying OXA-carbapenemase genes were found among hospital surfaces in Bangladesh in 2021^23^. In this study, however, *E. hormaechei*, *Pseudomonas* spp. and *Shewanella putrefaciens* were identified as the bacterial species carrying *bla*_OXA-48_-like genes in BK. Whilst we did detect *A. baumannii* (7.4%), this was mostly recovered from HSS collected in RU (ST52 carrying *bla*_NDM-1_), but none from Bangladesh. The present work confirms that *bla*_OXA-48_-like genes are widespread in African and South-Asian countries. Contrary to results from a hospital surface screen of multiple wards in a hospital in Ghana^1^, we showed a lower prevalence of *bla*_NDM_ (*n* = 338/6290 samples, 5.4%). Nevertheless, the prevalence of this gene, particularly in Pakistan (*n* = 162/1033, 15.7%), was in line with a study from several hospitals in the same country^41^. *bla*_KPC_ is not common in Africa or South Asia^7,26^, which this study also confirmed; however, *bla*_KPC_ was detected from neonatal cots in Tanzania in 2021^17^ and *bla*_KPC_ *K. pneumoniae* was found on pillows and the floor in three hospitals in Bangladesh^42^.

Limitations of this study include a lack of consistency in swabbing areas, as their descriptions were, on occasions, imprecise and the samples varied across each hospital. Thus, we were cautious with data interpretation and did not present correlation data between GNB growth and/or ARG prevalence per surface per hospital or between surface type/category and bacterial species due to sampling limitations. A second limitation was not grouping swab locations to reduce the testing size, which resulted in a total of 309 different surfaces, causing difficulties when comparing across countries and hospitals. Thirdly, there were differences in the number of samples collected across hospital sites and time, as we did not confirm the exact number/type of samples per month. Moreover, there was a lack of written information accompanying some samples—not all the samples were well-described. Information related to cleaning and disinfection practices in each hospital site was not collected, which might explain some of the results obtained; PCR screening for other genes would have also given us a broader view of potential resistance to other antibiotics available in LMICs but not commonly used due to economic resources or accessibility.

To summarise, we have shown that ESBL- and carbapenemase-producing *Enterobacterales* were most prevalent in samples collected near the sink drain. Transmission events occurred across patient care equipment and environmental surfaces of the hospital wards, and worryingly, there was evidence that the same strains have caused neonatal sepsis. Moreover, and of particular concern, is the high prevalence of antibiotic resistance determinants and the diversity of ARG-containing bacteria among surfaces within the hospital facilities, presenting an increasing threat to the patients. Future work to determine the relative risk of inborn neonates developing sepsis with specific bacterial strains that are colonising HSS is warranted to fully understand the impact of bacterial transmission events across the wards in neonatal sepsis. Our results further emphasise the extent of hospital surface contamination with bacteria carrying multiple ARG in LMICs, calling for an urgent assessment of improved IPC practice compliance and tailored guidelines for each hospital site.

## Methods

### Settings, ethics, participants, and study design

The BARNARDS network included Bangladesh, Chittagong Maa-O-Shishu Hospital, Chattogram (BC), and Kumudini Women’s Medical College, Mirzapur (BK); Ethiopia, St. Paul’s Hospital Millennium Medical College, Addis Ababa (ES); India, Division of Bacteriology, ICMR-National Institute of Cholera and Enteric Diseases Beliaghata and Institute of Post-Graduate and Medical Education and Research, Kolkata (IN); Nigeria, National Hospital Abuja (NN), Wuse District Hospital, Abuja (NW), and Murtala Mohammad Specialist Hospital, Kano (NK); Pakistan, Pakistan Institute of Medical Sciences (PP) and Bhara Kahu Rural Health Centre, Bhara Kahu (PC); Rwanda, University Central Hospital of Kigali, Kigali (RU) and Kabgayi Hospital, Kabgayi (RK); South Africa, Tygerberg Hospital, Cape Town (ZAT). HSS were not collected in India or one of the hospitals in Pakistan (PC). The hospital site abbreviation names are used throughout this article; however, the country name is used when the results are applicable to all hospitals within that country.

Standard operating procedures (SOPs) were designed and adhered to throughout the network (https://www.ineosoxford.ox.ac.uk/research/areas-of-focus/amr-burden/barnards), and ethical approval was obtained from local ethics committees prior to the start of the study.

From November 2015 until January 2018, HSS were collected from different surfaces (Supplementary Data 1) within different wards (i.e. maternity and neonatal intensive care units). Frequently, information regarding wards was incomplete, thus, only the surface but not their location within the hospitals was considered for analysis. Samples from environmental surfaces and patient care equipment in hospital settings were collected with charcoal swabs and stored at 4 °C until transported to the UK under UN3373 conditions.

### HSS processing

**Fig. 5:**
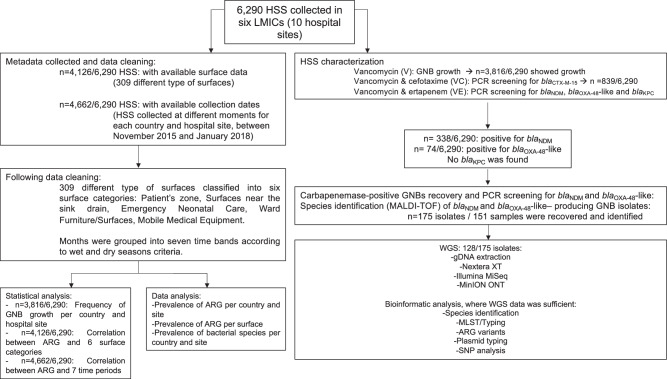
Workflow of sample collection and processing. Diagram detailing the total number of hospital surface swabs (HSS) collected, showing Gram-negative bacteria (GNB) growth, and screened for the presence of *bla*_CTX-M-15_, *bla*_NDM_, *bla*_KPC_ and *bla*_OXA-48-like_ antimicrobial resistance genes (ARGs), the number of GNB isolates recovered carrying carbapenemase genes, the number of isolates characterised by whole genome sequencing (WGS) and, where WGS data was sufficient, bioinformatic analysis was performed. Isolates for WGS were chosen after culture on VE (vancomycin, ertapenem) agar. Recoverable isolates after −80 °C preservation were selected for gDNA extraction and WGS. Data cleaning is also detailed; where data regarding hospital surfaces and collection dates was available, statistical analysis and data analysis were performed.

HSS were streaked on three chromogenic agar media plates (Liofilchem®, Italy) supplemented with vancomycin (10 mg/L) to promote the growth of GNB, vancomycin and cefotaxime (VC, 10 and 1 mg/L, respectively) to select for ESBL producers, and vancomycin and ertapenem (VE, 10 and 2 mg/L, respectively) to select for carbapenemase producers^7^. VC plates were tested for the presence of *bla*_CTX-M-15_ by PCR and VE plates were tested for *bla*_NDM_, *bla*_OXA-48_-like and *bla*_KPC_ by multiplex-PCR. All bacterial cultures were preserved in TS/72 cryogenic beads (Technical Service Consultants, UK) and stored at −80 °C. When VE bacterial growth yielded multiplex-PCR positive results, phenotypically distinct bacterial colonies were isolated and screened for the carbapenemase genes in the study by multiplex-PCR. Those isolates with a positive result were identified by MALDI-TOF MS (Bruker Daltonik GmbH, Coventry, UK) and preserved as detailed above. As per the BARNARDS protocol, due to the high prevalence of *bla*_CTX-M-15_, we did not scrutinise samples for *bla*_CTX-M-15_ positive isolates. The study workflow and dataset are summarised in Fig. 5. ARG prevalence resulting from PCR screening was represented in Coloured maps, which were created using MapChart (https://www.mapchart.net).

### Whole genome sequencing (WGS)

WGS was carried out on GNB isolates positive for at least one of the carbapenemase genes. Briefly, and as detailed by Sands et al.^26^, gDNA was extracted using the QIAmp DNA mini kit (Qiagen, Germany) on the QIAcube platform (Qiagen, Germany) and quantified using the Qubit fluorometer 4.0. Short-read sequencing libraries were prepared using the Nextera XT v2 kit and paired-end sequenced on an Illumina MiSeq using the V3 chemistry to generate fragment lengths up to 300 bp (600 cycles). Long reads were prepared using Oxford Nanopore Technology (ONT) and libraries were generated using the 96-Rapid Barcoding Kit (SQK-RBK110.96; ONT). Sequencing was performed using MinION flow cells (R9.4.1) for a running time of 72 h within MinKnow.

### Bioinformatic analysis

To determine whether bacterial transmission events occurred within the hospital wards and/or neonatal sepsis, bioinformatic analysis was performed as detailed by Sands K et al.^26^. ONT FAST5 reads were base called using Guppy v5.0.11 and NVIDIA V100 GPUs. Following QC on short reads (Illumina) and long reads (ONT) as described by Boostrom et al.^43^, reads were assembled using Unicycler (v0.4.9). Genome assembly metrics were generated using QUAST (v.5.2.0)^44^ and bacterial species were identified using Pathogenwatch^45^. Multilocus sequence typing (MLST), characterisation of genes conferring resistance to antibiotics and/or disinfectants genes and plasmid genomic profiles were performed using ABRicate (v0.9.7)^46^ and associated databases: NCBI^47^, PlasmidFinder^48^ and CARD^49^. Previously undefined alleles and sequence type (ST) profiles were submitted to Enterobase, BIGSbd and PubMLST for assignment^50^. Genomes were annotated using Prokka (v1.14.5)^51^. Following initial WGS analysis, four or more isolates with the matching ST/species from a single hospital site were considered a potential cluster and were subject to SNP analysis using snippy (v4.6.0)^52^ with BWA and freebayes mapping the reads and calling variants --min cov 20. To maximise SNP calling, a high-quality internal reference was used^53^. A pairwise SNP matrix was performed converting a FASTA alignment to an SNP distance matrix using Snp-dists (v0.8.2)^54^. Adobe Illustrator v26.5 was used to generate the isolate-relatedness timeline figure. The Sankey diagram was created using Sankeymatic.com.

For plasmid analysis for evidence of transmission between bacterial isolates/species, the contig containing the carbapenemase variant and plasmid was analysed using the Mauve aligner^55^ within Geneious (v2023.2.1). The size of the plasmid and whether the assembler theoretically denoted the contig as circular and complete were recorded. Following Mauve alignment, the plasmid sequences were annotated using Prokka (v1.14.5) and further annotated using a reference plasmid from a BLAST search within Geneious. Visualisations of annotated aligned plasmids were performed within Geneious. Further similarity matrices were generated using mash dist (v2.2)^56^ to estimate the genetic similarity between plasmid sequences. Sequence alignments were performed within Geneious using the MAFFT aligner plugin.

### Data cleaning and statistical analysis

To analyse the frequency of GNB growth in hospital surfaces, GNB growth on plates supplemented with vancomycin was examined, and the total number of processed samples was used as a denominator for the prevalence of assessed ARGs. HSS was collected from different surfaces (Supplementary Data 1) and included both environmental surfaces and patient care equipment in hospital settings, which were classified into six different categories (Supplementary Data 7) based on WHO and CDC IPC guidelines and previous publications^4,13,18,57^, following data cleaning. In this study, environmental surfaces included the hands of healthcare workers, which were categorised into the patient’s zone as they can act as a source of transmission^4,58,59^. The category of patient care equipment was defined as medical equipment or mobile medical equipment, categories that include non-critical, semi-critical and noncritical patient care equipment. The six final categories were: surfaces from the patient’s zone (immediate environment), surfaces near the sink drain (including sink basin, faucet, faucet handles, and surrounding countertop)^60,61^, emergency neonatal care, ward furniture/surfaces, mobile medical equipment, and medical equipment. Due to unequal sample size (Table 2) as well as sample variety (Supplementary Data 1), statistical analyses were performed using the total number of samples included in each surface category and not per country/hospital. Furthermore, following data cleaning, the HSS collection dates were classified into seven-time bands (Supplementary Table 4) according to Climate Change Knowledge Portal (World Bank Group)^62^. Statistical analysis to study the ARG prevalence was performed considering HSS collected per time band as a denominator, inclusive of all countries and hospitals. No seasonal analysis per country was performed.

ARG frequencies per hospital, country and surface category and corresponding figures were assessed. RStudio ggplot2 package was used for figure creation (RStudio version 4.3.0 (2023-04-21) -- “Already Tomorrow”), and IBM SPSS Statistics (Version 25.0.0.1) (190) was used to perform the statistical analyses. Statistical analyses were performed to determine whether certain surfaces were at greater risk of colonisation with β-lactamase-producing bacteria. The relationship between ARG and a surface category, as well as the frequency of GNB colonisation of hospital surfaces among countries and hospital sites, and over the timeline, were analysed to obtain counts and percentages. Chi-Squared tests were conducted to test the independence of the variables on contingency tables to establish if the overall differences in frequencies between GNB colonisation and ARG prevalence over countries and surfaces were statistically significant at the *P* < 0.05 level. When such differences were seen, the individual proportions for each of the countries and surfaces were examined and compared to determine where the main differences lay.

### Reporting summary

Further information on research design is available in the Nature Portfolio Reporting Summary linked to this article.

### Supplementary information

Supplementary Information

Peer Review File

Description of Additional Supplementary Files

Supplementary data 1

Supplementary data 2

Supplementary data 3

Supplementary data 4

Supplementary data 5

Supplementary data 6

Supplementary data 7

Supplementary data 8

Reporting Summary

### Source data

Source Data

## Data Availability

Databases used for in silico analysis in this work are PlasmidFinder, CARD, Enterobase, and PubMLST. Coloured maps in the paper were created using MapChart (https://www.mapchart.net). The dataset generated in this study is deposited in the Figshare repository (10.6084/m9.figshare.23790360). Source data are provided with this paper; available as Supplementary Data files or provided as a Source Data file. Genomes are available in the NCBI database under BioProject number PRJNA971772 (and accession codes/accessible links are provided in Supplementary Data 8). The plasmid analysis data generated in this study for evidence of transmission is available in Supplementary figures. Source data are provided with this paper.
