# Supplementary material for: Colonisation of hospital surfaces from low- and middle-income countries by extended spectrum β-lactamase- and carbapenemase-producing bacteria
Source: Nat Commun. 2024 Mar 29;15:2758. doi: 10.1038/s41467-024-46684-z (PMC10980694; doi:10.1038/s41467-024-46684-z)
Supplement: Supplementary file 1 — Supplementary Information [file 41467_2024_46684_MOESM1_ESM.pdf]

Concomitant ARG carriage	N samples	% samples
$bla_{CTX\text{-}M\text{-}15}$ and bla_{NDM}	116	1.84
$bla_{CTX\text{-}M\text{-}15}$ and $bla_{OXA\text{-}48}$-like	46	0.73
bla_{NDM} and $bla_{OXA\text{-}48}$-like	42	0.67

Supplementary Table 1. Frequency of samples showing concomitant antimicrobial resistance gene (ARG) carriage.

	N (%) $bla_{CTX\text{-}M\text{-}15}$	N (%) bla_{NDM}	N (%) bla_{KPC}	N (%) $bla_{OXA\text{-}48}$-like	Total HSS collected in each country and hospital site
**B**	122 (11.9)<br>BC: 60 (14.6)<br>BK: 62 (10.1)	149 (14.6)<br>BC: 43 (10.5)<br>BK: 106 (17.3)	0 (0)	7 (0.7)<br>BC: 5 (1.2)<br>BK: 2 (0.3)	B: 1,024<br>BC: 410<br>BK: 614
**E (ES)**	ES: 31 (12.3)	1 (0.4)	0 (0)	4 (1.6)	253
**N**	267 (17.0)<br>NK: 181 (38.3)<br>NN: 41 (5.9)<br>NW: 45 (11.3)	55 (3.5)<br>NK: 43 (9.1)<br>NN: 11 (1.6)<br>NW: 1 (0.3)	0 (0)	12 (0.8)<br>NK: 6 (1.3)<br>NN: 5 (0.7)<br>NW: 1 (0.3)	1,566<br>NK: 472<br>NN: 695<br>NW: 399
**P (PP)**	PP: 162 (15.7)	118 (11.4)	0 (0)	32 (3.1)	1,033
**R**	204 (22.0)<br>RK: 114 (24.4)<br>RU: 90 (19.6)	13 (1.4)<br>RK: 3 (0.6)<br>RU: 10 (2.2)	0 (0)	8 (0.9)<br>RK: 5 (1.1)<br>RU: 5 (0.7)	927<br>RK: 467<br>RU: 460
**SA (ZAT)**	ZAT: 53 (3.6)	2 (0.1)	0 (0)	11 (0.7)	1,487
				TOTAL	6,290

Supplementary Table 2. Prevalence of antimicrobial resistance genes (ARGs) per country and hospital site, over the number of hospital surface swabs (HSS) collected per country and hospital site. The total number of samples collected per country and per hospital site were used as denominators to calculate the prevalence of each ARG per country and per hospital site.
Abbreviations for BARNARDS hospitals detailed in methods section of the paper.

a)

Bacterial species and n of isolates carrying carbapenemases recovered from HSS	
Achromobacter insolitus	1
Achromobacter spanius	1
Acinetobacter baumannii	13
Acinetobacter bereziniae	1
Acinetobacter nosocomialis	1
Enterobacter asburiae	2
Enterobacter cloacae	8
Enterobacter hormaechei	38
Enterobacter ludwigii	2
Enterobacter roggenkampii	3
Escherichia coli	3
Klebsiella michiganensis	4
Klebsiella oxytoca	1
Klebsiella pneumoniae	46
Klebsiella quasipneumoniae	2
Kosakonia cowanii	1
Leclercia adecarboxylata	11
Pseudomonas aeruginosa	6
Pseudomonas asiatica	1
Pseudomonas mendocina	3
Pseudomonas putida	2
Pseudomonas stutzeri	2
Serratia marcescens	12
Shewanella putrefaciens	2
Shewanella xiamenensis	2
Stenotrophomonas acidaminiphila	1
Stenotrophomonas maltophilia	3
Unidentified	3
	175

b)

Bacterial species	n and % of isolates carrying carbapenemases per surface category						
	Surfaces near the sink drain	Mobile medical equipment	Ward furniture/surfaces	Emergency neonatal care	Medical equipment	Patient's zone	NA
Achromobacter insolitus	0(0%)	0(0%)	0(0%)	0(0%)	0(0%)	0(0%)	1(100%)
Achromobacter spanius	0(0%)	1(100%)	0(0%)	0(0%)	0(0%)	0(0%)	0(0%)
Acinetobacter baumannii	0(0%)	2(15.38%)	1(7.69%)	4(30.77%)	0(0%)	3(23.08%)	3(23.08%)
Acinetobacter bereziniae	1(100%)	0(0%)	0(0%)	0(0%)	0(0%)	0(0%)	0(0%)
Acinetobacter nosocomialis	0(0%)	0(0%)	1(100%)	0(0%)	0(0%)	0(0%)	0(0%)
Enterobacter asburiae	0(0%)	1(50%)	1(50%)	0(0%)	0(0%)	0(0%)	0(0%)
Enterobacter cloacae	1(12.5%)	0(0%)	0(0%)	1(12.5%)	3(37.5%)	2(25%)	1(12.5%)
Enterobacter hormaechei	4(10.53%)	1(2.63%)	6(15.79%)	6(15.79%)	5(13.16%)	2(5.26%)	14(36.84%)
Enterobacter ludwigii	0(0%)	0(0%)	0(0%)	0(0%)	0(0%)	0(0%)	2(100%)
Enterobacter roggenkampii	0(0%)	0(0%)	1(33.33%)	0(0%)	0(0%)	2(66.67%)	0(0%)
Escherichia coli	0(0%)	0(0%)	0(0%)	1(33.33%)	1(33.33%)	1(33.33%)	0(0%)
Klebsiella michiganensis	0(0%)	1(25%)	0(0%)	0(0%)	2(50%)	0(0%)	1(25%)
Klebsiella oxytoca	1(100%)	0(0%)	0(0%)	0(0%)	0(0%)	0(0%)	0(0%)
Klebsiella pneumoniae	1(2.17%)	4(8.7%)	2(4.35%)	10(21.74%)	8(17.39%)	11(23.91%)	10(21.74%)
Klebsiella quasipneumoniae	1(50%)	0(0%)	0(0%)	0(0%)	1(50%)	0(0%)	0(0%)
Kosakonia cowanii	0(0%)	0(0%)	0(0%)	0(0%)	0(0%)	1(100%)	0(0%)
Leclercia adecarboxylata	1(9.09%)	2(18.18%)	0(0%)	3(27.27%)	0(0%)	4(36.36%)	1(9.09%)
Pseudomonas aeruginosa	1(16.67%)	0(0%)	1(16.67%)	0(0%)	1(16.67%)	0(0%)	3(50%)
Pseudomonas asiatica	0(0%)	0(0%)	1(100%)	0(0%)	0(0%)	0(0%)	0(0%)
Pseudomonas mendocina	1(33.33%)	0(0%)	0(0%)	0(0%)	0(0%)	0(0%)	2(66.67%)
Pseudomonas putida	0(0%)	0(0%)	0(0%)	0(0%)	0(0%)	0(0%)	2(100%)
Pseudomonas stutzeri	0(0%)	0(0%)	0(0%)	1(50%)	1(50%)	0(0%)	0(0%)
Serratia marcescens	4(33.33%)	0(0%)	0(0%)	0(0%)	2(16.67%)	0(0%)	6(50%)
Shewanella putrefaciens	1(50%)	0(0%)	0(0%)	0(0%)	0(0%)	0(0%)	1(50%)
Shewanella xiamenensis	1(50%)	0(0%)	0(0%)	0(0%)	1(50%)	0(0%)	0(0%)
Stenotrophomonas acidaminiphila	0(0%)	0(0%)	0(0%)	0(0%)	0(0%)	0(0%)	1(100%)
Stenotrophomonas maltophilia	0(0%)	0(0%)	1(33.33%)	0(0%)	0(0%)	0(0%)	2(66.67%)
Unidentified	0(0%)	1(33.33%)	0(0%)	0(0%)	0(0%)	0(0%)	2(66.67%)

c)

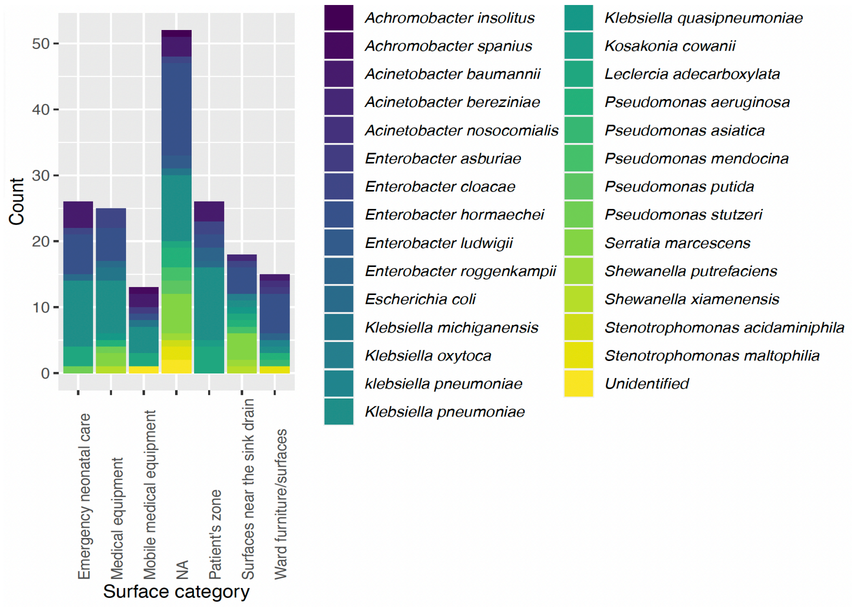

Supplementary Table 3. n (%) of bacterial species and isolates per surface category carrying *bla* $_{NDM}$ and *bla* $_{OXA-48}$-like according to PCR screening and identification by MALDI-TOF MS. A total of 175 bacterial isolates from 27 bacterial species identified by MALDI-TOF MS (and 3 "unidentified" isolates) were recovered. Abbreviations for BARNARDS hospitals detailed in methods section of the paper.

a)Bacterial species and n of isolates carrying carbapenemases recovered from HSS identified by MALDI-TOF MS. b)Prevalence of the different bacterial species identified carrying carbapenemases is represented per surface category. The percentages shown were calculated by dividing the number of bacterial species isolates found in a specific surface category (n in table b)) by the total of isolates recovered from HSS (a). NA represents those isolates recovered from HSS, which lacked of surface metadata associated. c)Stacked bar chart created with RStudio ggplot2 package to visualise the data from a) and b) (RStudio version 4.3.0 (2023-04-21) -- "Already Tomorrow"). Source data are provided in Tables a) and b) from this Table 3.

a)

Country	Dry season	Wet season
Bangladesh	December-February (cold temperatures)	April-September or October (warm temperatures)
Ethiopia	October-December (cold temperatures)	June-September / February-May: less rainfall
Nigeria	November-March (March is the hottest month)	April-October / March-October /March-November (August is the coldest month and June is the wettest)
Pakistan	December-May (cold and hot temperatures)	June-November
Rwanda	June-august / December-February	March-May / September-November
South Africa (majority of the country but differences for Cape Town)	June-August	December-February / November-March: highest rainfall (warm temperatures)<br>Cape Town: May-August rainy season (winter)

b)

Time band	Number of HSS included per month	Number of HSS included per banding	Number of HSS in Banglades h	Number of HSS in Ethiopia	Number of HSS in Nigeria	Number of HSS in Pakistan	Number of HSS in Rwanda	Number of HSS in South Africa
**November 2015**	37	309	2	0	35	0	0	0
**December 2015**	72		27	0	45	0	0	0
**January 2016**	109		32	0	45	10	22	0
**February 2016**	91		26	0	45	20	0	0
**March 2016**	115	693	37	0	45	33	0	0
**April 2016**	146		29	0	55	40	22	0
**May 2016**	232		32	0	70	59	46	25
**June 2016**	200		15	0	45	64	23	53
**July 2016**	276	819	47	0	45	60	46	77
**August 2016**	198		64	0	61	55	0	20
**September 2016**	171		41	0	44	59	23	3
**October 2016**	174		39	0	20	55	23	36
**November 2016**	191	1014	50	0	35	56	45	5
**December 2016**	226		34	0	29	46	66	51
**January 2017**	254		30	0	21	55	23	125
**February 2017**	343		42	0	25	40	23	213
**March 2017**	186	688	32	0	25	61	68	0
**April 2017**	142		45	0	55	20	22	0
**May 2017**	156		62	0	43	5	46	0
**June 2017**	204		64	0	57	15	68	0
**July 2017**	300	892	47	0	186	0	68	0
**August 2017**	143		31	42	35	13	22	0
**September 2017**	229		48	35	72	5	69	0
**October 2017**	220		60	55	34	15	56	0
**November 2017**	169	247	41	45	25	5	53	0
**December 2017**	68		2	61	0	5	0	0
**January 2018**	10		0	10	0	0	0	0
TOTAL		4,662	979	248	1197	796	834	608
			4,662					

Supplementary Table 4. Hospital surface swabs (HSS) collection dates classified into seven time bands, to study the antimicrobial resistance genes (ARGs) prevalence during the study timeline (November 2015 – January 2018).

a) Dry and wet seasons per country (some of them showing information on temperatures) based on the Climate change knowledge portal (World Bank Group). Differences between certain parts of a country exist, thus, the seasons are described for most part of a country. Moreover, dry and wet seasons occur during opposite months, or more than one dry/wet season exist for some countries, and the duration of a season is different in each country. This table was only taken into consideration to stablish seven time bands according to the months included per season, but no seasonal analysis per country was performed because of all the mentioned differences between countries. b) Timeline classification into seven time bands and number of HSS collected per country during the study timeline. Information regarding collection dates was available for a total of 4,662 HSS, which were considered for the analysis of ARG prevalence during timeline.

Country	Forename	Surname	Position	Affiliation
Bangladesh	Samir	Saha	PI	Chittagong Medical College Hospital, Bangladesh
Bangladesh	Maksuda	Islam	scientist	Chittagong Medical College Hospital, Bangladesh
Bangladesh	Zabed	Bin-Ahmed	scientist	Chittagong Medical College Hospital, Bangladesh
Bangladesh	Wazir	Ahmed	clinician	Chittagong Medical College Hospital, Bangladesh
Bangladesh	Taslima	Begum	clinician	Chittagong Medical College Hospital, Bangladesh
Bangladesh	Mitu	Chowdhury	scientist	Chittagong Medical College Hospital, Bangladesh
Bangladesh	Shaila	Sharmin	scientist	Chittagong Medical College Hospital, Bangladesh
Bangladesh	Chumki	Rani Dey	Research nurse	Chittagong Medical College Hospital, Bangladesh
Bangladesh		Uttam	scientist	Chittagong Medical College Hospital, Bangladesh
Bangladesh	Abdul	Matin	clinician	Kumudini Women's Medical College, Bangladesh
Bangladesh	Sowmitra Ranja	Chakraborty	scientist	Kumudini Women's Medical College, Bangladesh
Bangladesh	Sadia	Tasmin	clinician	Kumudini Women's Medical College, Bangladesh
Bangladesh	Dipa	Rema	Research nurse	Kumudini Women's Medical College, Bangladesh
Bangladesh	Rashida	Khatun	Research nurse	Kumudini Women's Medical College, Bangladesh
Bangladesh	Liza	Nath	Research nurse	Kumudini Women's Medical College, Bangladesh
Ethiopia	Nigatu	Balkachew	clinician	Department of Ob-Gyn, Saint Paul's Hospital Millennium Medical College
Ethiopia	Delayehu	Bekele	Co-PI	Obstetrics and Gynaecology, Saint Paul's Hospital Millennium Medical College
Ethiopia	Katherine	Schaughency	Data manager	Saint Paul's Hospital Millennium Medical College
Ethiopia	Semaria	Solomon	laboratory	Department of Microbiology, Immunology and Parasitology, Saint Paul's Hospital Millennium Medical College
Ethiopia	Zenebe	Gebreyohanes	laboratory	Department of Microbiology, Immunology and Parasitology, Saint Paul's Hospital Millennium Medical College
Ethiopia	Rozina	Ambachew	laboratory	Department of Microbiology, Immunology and Parasitology, Saint Paul's Hospital Millennium Medical College
Ethiopia	Oludare	Odumade	clinician	Department of Pediatrics, Boston Children's Hospital Harvard Medical School
Ethiopia	Misgana	Haileselassie	nurse	Saint Paul's Hospital Millennium Medical College
Ethiopia	Grace	Chan	PI	Paediatrics, Harvard Medical School
Ethiopia	Abigail	Russo	Project manager	Global Health Research Program Manager, Boston Children's Hospital
Ethiopia	Redeat	Workneh	Project manager	Department of Pediatrics and Child Health, Saint Paul's Hospital Millennium Medical College
Ethiopia	Gesit	Metaferia	clinician	Department of Pediatrics, Saint Paul's Hospital Millennium Medical College
Ethiopia	Mahlet	Abayneh		Saint Paul's Hospital Millennium Medical College
Ethiopia	Yahya Zekaria	Mohammed		Birhan for Mothers and Children Program, Saint Paul's Hospital Millennium Medical College
Ethiopia	Tefera	Biteye		Birhan for Mothers and Children Program, Saint Paul's Hospital Millennium Medical College
Ethiopia	Alula	Teklu	scientist	Saint Paul's Hospital Millennium Medical College
Ethiopia	Wendimagegn	Gezahegn	clinician	Department of Pediatrics, Saint Paul's Hospital Millennium Medical College
India	Partha Sarathi	Chakravorty	clinician	Obstetrics & Gynecology, IPGMER & SSKM Hospital
India	Anuradha	Mukherjee	clinician	Division of Bacteriology, ICMR-National Institute of Cholera and Enteric Diseases,Kolkata-700010,India
India	Ranjan Kumar	Nandy	Co-PI	Scientist-F, Division of Bacteriology, ICMR-National Institute of Cholera and Enteric Diseases
India	Samarpan	Roy	Data entry operator	ICMR-National Institute of Cholera and Enteric Diseases
India	Anuradha	Sinha	Deputy manager	R&D, Zydus Cadila
India	Sharmi	Naha	laboratory	ICMR-SRF, Division of Bacteriology, ICMR-National Institute of Cholera and Enteric Diseases
India	Sukla Saha	Malakar	laboratory	ICMR-National Institute of Cholera and Enteric Diseases
India	Siddhartha	Bose	laboratory	Medical Laboratory Technologist, Swasthya Bhawan
India	Monaki	Majhi	laboratory	Baruipur SD Hospital
India	Subhasree	Sahoo	nurse	
India	Putul	Mukherjee	nurse	
India	Sumitra Kumari	Routa	nurse	
India	Chaitali	Nandi	nurse	
India	Sulagna	Basu	PI	Scientist F, Division of BacteriologyICMR-National Institute of Cholera and Enteric Diseases,
India	Bijan	Saha	scientist	Neonatology, IPGMER & SSKM Hospital
India	Pinaki	Chattopadhyay	scientist	Neonatal Microbiology, IPGMER & SSKM Hospital
Nigeria	Fatima Zara Isa	Modibbo	clinician	54gene
Nigeria	Stella	Uwaezuoke	clinician	Federal Medical Centre Jabi, Abuja, Nigeria
Nigeria	Dilichukwu	Meduekwe	clinician	Wuse District Hospital, Nigeria
Nigeria	Khairiyya	Muhammad	laboratory	National Hospital Abuja, Nigeria
Nigeria	Queen	Nsude	laboratory	Wuse District Hospital, Nigeria
Nigeria	Ifeoma	Ukeh	laboratory	National Hospital Abuja, Nigeria
Nigeria	Mary-Joe	Okenu	laboratory	National Hospital Abuja, Nigeria
Nigeria	Akpulu	Chinenye	laboratory	National Hospital Abuja, Nigeria
Nigeria	Samuel	Yakubu	laboratory	National Hospital Abuja, Nigeria
Nigeria	Vivian	Asunugwo	laboratory	Wuse District Hospital, Nigeria
Nigeria	Folake	Aina	NYS Corp Member	National Hospital Abuja, Nigeria
Nigeria	Isibong	Issy	NYS Corp Member	National Hospital Abuja, Nigeria
Nigeria	Dolapo	Adekeye	NYS Corp Member	National Hospital Abuja, Nigeria
Nigeria	Adiele	Eunice	nurse	Wuse District Hospital, Nigeria
Nigeria	Abdulmlik	Amina	nurse	Wuse District Hospital, Nigeria
Nigeria	R	Oyewole	nurse	National Hospital Abuja, Nigeria
Nigeria	I	Oloton	nurse	National Hospital Abuja, Nigeria
Nigeria	BC	Nnaji	nurse	National Hospital Abuja, Nigeria
Nigeria	M	Umejiego	nurse	National Hospital Abuja, Nigeria
Nigeria	PN	Anoke	nurse	National Hospital Abuja, Nigeria
Nigeria	S	Adebayo	nurse	National Hospital Abuja, Nigeria
Nigeria	GO	Abegunrin	nurse	National Hospital Abuja, Nigeria
Nigeria	OB	Omotosho	nurse	National Hospital Abuja, Nigeria
Nigeria	R	Ibrahim	nurse	National Hospital Abuja, Nigeria
Nigeria	B	Igwe	nurse	National Hospital Abuja, Nigeria
Nigeria	M	Abroko	nurse	National Hospital Abuja, Nigeria
Nigeria	K	Balami	nurse	National Hospital Abuja, Nigeria
Nigeria	L	Bayem	nurse	National Hospital Abuja, Nigeria
Nigeria	C	Anyanwu	nurse	National Hospital Abuja, Nigeria
Nigeria	H	Haruna	nurse	National Hospital Abuja, Nigeria
Nigeria	J	Okike	nurse	National Hospital Abuja, Nigeria
Nigeria	K	Goroh	nurse	National Hospital Abuja, Nigeria
Nigeria	M	Boi-Sunday	nurse	National Hospital Abuja, Nigeria
Nigeria	Augusta	Ugafor	nurse	National Hospital Abuja, Nigeria
Nigeria	Maryam	Makama	nurse	National Hospital Abuja, Nigeria
Nigeria	Kaniba	Ndukwe	nurse	National Hospital Abuja, Nigeria
Nigeria	Anastesia	Odama	nurse	National Hospital Abuja, Nigeria
Nigeria	Hadiza	Yusuf	nurse	National Hospital Abuja, Nigeria
Nigeria	Patience	Wachukwu	nurse	National Hospital Abuja, Nigeria
Nigeria	Kachalla	Yahaya	nurse	National Hospital Abuja, Nigeria
Nigeria	Titus	Kalade Colson	nurse	National Hospital Abuja, Nigeria
Nigeria	Mercy	Kura	nurse	National Hospital Abuja, Nigeria
Nigeria	Damilola	Orebiyi	nurse	National Hospital Abuja, Nigeria
Nigeria	Kenneth C.	Iregbu	PI	National Hospital Abuja, Nigeria
Nigeria	Chukwuemeka	Mmadueke	clinician	Wuse District Hospital, Nigeria
Nigeria	Lamidi	Audu	clinician	National Hospital Abuja, Nigeria
Nigeria	Nura	Idris	clinician	Director Kano State Health Trust Fund
Nigeria	Safiya	Gambo	clinician	Murtala Muhammad Specialist Hospital, Nigeria
Nigeria	Jamila	Ibrahim	clinician	Department of paediatric Muhammad Abdullahi Wase Teaching Hospital
Nigeria	Edwin	Precious	scientist	Department of Microbiology Aminu Kano Teaching Hospital
Nigeria	Ashiru	Hassan		Murtala Muhammad Specialist Hospital, Nigeria
Nigeria	Shamsudden	Gwadabe	scientist	Department of Physiology NorthWest University Kano

Nigeria	Adeola	Adeleye Falola	scientist	54gene
Nigeria	Muhammad	Aliyu	scientist	Murtala Muhammad Specialist Hospital, Nigeria
Nigeria	Amina	Ibrahim	scientist	Murtala Muhammad Specialist Hospital, Nigeria
Nigeria	Aisha Sani	Mukaddas	scientist	Murtala Muhammad Specialist Hospital, Nigeria
Nigeria	Rashida Yakubu	Khalid	scientist	Murtala Muhammad Specialist Hospital, Nigeria
Nigeria	Fatima Ibrahim	Alkali	scientist	Murtala Muhammad Specialist Hospital, Nigeria
Nigeria	Maryam Yahaya	Muhammad	scientist	Murtala Muhammad Specialist Hospital, Nigeria
Nigeria	Fatima Mohamm	Tukur	scientist	Murtala Muhammad Specialist Hospital, Nigeria
Nigeria	Surayya Mustap	Muhammad	scientist	Department of Biotechnology Federal University Dutse
Nigeria	Adeola	Shittu	scientist	Murtala Muhammad Specialist Hospital, Nigeria
Nigeria	Murjanatu	Bello	scientist	Murtala Muhammad Specialist Hospital, Nigeria
Nigeria	Muhammad Ab	Hassan	scientist	Murtala Muhammad Specialist Hospital, Nigeria
Nigeria	Fatima Habib	Sa ad	scientist	Murtala Muhammad Specialist Hospital, Nigeria
Nigeria	Aishatu	Kassim	scientist	Murtala Muhammad Specialist Hospital, Nigeria
Pakistan	Haider	Shirazi	Co-PI	Neonatology Department, Pakistan Institute of Medical Sciences
Pakistan	Adil	Muhammad	laboratory	Department of Microbiology, Quaid-i-Azam University, Islamabad 45320, Pakistan
Pakistan	Rabaab	Zahra	PI	Department of Microbiology, Quaid-i-Azam University, Islamabad 45320, Pakistan
Pakistan	Syed Najeeb	Ullah	scientist	Department of Microbiology, Quaid-i-Azam University, Islamabad 45320, Pakistan
Pakistan	Muhammad	Hilal Jan	project manager	Department of Microbiology, Quaid-i-Azam University, Islamabad 45320, Pakistan
Pakistan	Rubina	Kamran	clinician	Pakistan Institute of Medical Sciences
Pakistan		Sajana	research nurse	Pakistan Institute of Medical Sciences
Pakistan	Jazba	Saeed	research nurse	Pakistan Institute of Medical Sciences
Pakistan	Noreen	Maqsood	research nurse	Pakistan Institute of Medical Sciences
Pakistan	Maria	Zafar	research nurse	Pakistan Institute of Medical Sciences
Pakistan	Saraeen	Sadiq	research nurse	Pakistan Institute of Medical Sciences
Pakistan	Sumble	Ahsan	research nurse	Pakistan Institute of Medical Sciences
Pakistan	Madiha	Tariq	scientist	Pakistan Institute of Medical Sciences
Pakistan	Sidra	Sajid	scientist	Pakistan Institute of Medical Sciences
Pakistan	Hasma	Mustafa	scientist	Pakistan Institute of Medical Sciences
Pakistan	Anees-ur	Rehman	local project manager	Pakistan Institute of Medical Sciences
Pakistan	Atif	Muhammad	local project manager	Pakistan Institute of Medical Sciences
Pakistan	Gahssan	Mehmood	local project manager	Pakistan Institute of Medical Sciences
Pakistan	Mahnoor	Nisar	local project manager	Pakistan Institute of Medical Sciences
Pakistan	Shermeen	Akif	local project manager	Pakistan Institute of Medical Sciences
Pakistan	Tahira	Yasmeen	local project manager	Pakistan Institute of Medical Sciences
Pakistan	Sabir	Nawaz	community coordinator	Pakistan Institute of Medical Sciences
Pakistan	Anam Shanal	Atta	community coordinator	Pakistan Institute of Medical Sciences
Pakistan	Mian	Laiq-ur-Rehma	community coordinator	Pakistan Institute of Medical Sciences
Pakistan	Robina	Kousar	community nurse	Pakistan Institute of Medical Sciences
Pakistan	Kalsoom	Bibi	community nurse	Pakistan Institute of Medical Sciences
Pakistan	Kosar	Waheed	community nurse	Pakistan Institute of Medical Sciences
Pakistan	Zainab	Majeed	community nurse	Pakistan Institute of Medical Sciences
Pakistan	Ayesha	Jalil	data support	Pakistan Institute of Medical Sciences
Rwanda	Espoir	Kajibwami	clinician	Kabgayi Hospital, Kabgayi, Gitarama, Rwanda
Rwanda	Aniceth	Rucogoza	laboratory	Microbiology Laboratory Director, National Reference Laboratory Rwanda.
Rwanda	Innocent	Nzabahimana	laboratory	The University Teaching Hostpial (CHUK), Kigali, Rwanda
Rwanda	Mazarati	Jean-Baptiste	PI	Head of Biomedical Services National Reference Laboratory, Kigali Rwanda
Rwanda	Lucie	Gaju	project manager	The University Teaching Hostpial (CHUK), Rwanda
Rwanda	Kankundiye	Riziki	nurse/scientist	The University Teaching Hostpial (CHUK), Kigali, Rwanda
Rwanda	Brigette	Uwamahoro	nurse/scientist	The University Teaching Hostpial (CHUK), Kigali, Rwanda
Rwanda	Rachel	Uwera	nurse/scientist	The University Teaching Hostpial (CHUK), Kigali, Rwanda
Rwanda	Eugenie	Nyiratuza	nurse/scientist	The University Teaching Hostpial (CHUK), Kigali, Rwanda
Rwanda	Kumwami	Muzungu	nurse/scientist	The University Teaching Hostpial (CHUK), Kigali, Rwanda
Rwanda	Violette	Uwitonze	nurse/scientist	Kabgayi Hospital, Kabgayi, Gitarama, Rwanda
Rwanda	Marie C	Horanimpundu	nurse/scientist	Kabgayi Hospital, Kabgayi, Gitarama, Rwanda
Rwanda	Francine	Nzeyimana	nurse/scientist	Kabgayi Hospital, Kabgayi, Gitarama, Rwanda
Rwanda	Prince	Mitima	nurse/scientist	Kabgayi Hospital, Kabgayi, Gitarama, Rwanda
South Africa	Angela	Dramowski	clinician	Department of Paediatrics and child health, Stellenbosch University, Stellenbosch, Cape Town
South Africa	Andrew	Whitelaw	Co-PI	Division of Medical Microbiology, Stellenbosch University, Tygerberg Hospital, Cape Town
South Africa	Lauren	Paterson	laboratory	Division of Medical Microbiology, Stellenbosch University, Tygerberg Hospital, Cape Town
South Africa	Mary	Frans	nurse	Tygerberg Hospital, Cape Town
South Africa	Marvina	Johnson	nurse	Tygerberg Hospital, Cape Town
South Africa	Eveline	Swanepoel	nurse	Tygerberg Hospital, Cape Town
South Africa	Zoleka	Bojana	nurse	Tygerberg Hospital, Cape Town
South Africa	Mieme	du Preez	nurse	Tygerberg Hospital, Cape Town
South Africa	Shaheen	Mehtar	PI	Faculty of Medicine and Health Sciences, Unit for Infection Prevention and Control, Stellenbosch University
South Africa	Andre	Bulabula	scientist	Faculty of Medicine and Health Sciences, Unit for Infection Prevention and Control, Stellenbosch University
The Netherlands	Feiyan	Liu	scientist	Leiden Academic Centre for Drug Research, Leiden University, Leiden, The Netherlands
The Netherlands	Johan GC	van Hasselt	scientist	Leiden Academic Centre for Drug Research, Leiden University, Leiden, The Netherlands
UK	Timothy	Walsh	PI	Institute of Infection and Immunity, School of Medicine, Cardiff University
UK	Kirsty	Sands	scientist	Institute of Infection and Immunity, School of Medicine, Cardiff University
UK	Maria	Carvalho	scientist	Institute of Infection and Immunity, School of Medicine, Cardiff University
UK	Rebecca	Milton	project manager	Institute of Infection and Immunity, School of Medicine, Cardiff University
UK	Kathryn	Thomson	scientist	Institute of Infection and Immunity, School of Medicine, Cardiff University
UK	Edward	Portal	scientist	Institute of Infection and Immunity, School of Medicine, Cardiff University
UK	Jordan	Mathias	scientist	Institute of Infection and Immunity, School of Medicine, Cardiff University
UK	Calie	Dyer	Data manager	Institute of Infection and Immunity, School of Medicine, Cardiff University
UK	Ana	Ferreira	scientist	Institute of Infection and Immunity, School of Medicine, Cardiff University
UK	Robert	Andrews	scientist	Institute of Infection and Immunity, School of Medicine, Cardiff University
UK	John	Watkins	scientist	Institute of Infection and Immunity, School of Medicine, Cardiff University
UK	David	Gillespie	scientist	Centre for Trials Research, Cardiff University
UK	Kerry	Hood	Co-PI	Centre for Trials Research, Cardiff University
UK	Katie	Taiyai	scientist	Centre for Trials Research, Cardiff University
UK	Nigel	Kirby	scientist	Centre for Trials Research, Cardiff University
UK	Maria	Nieto	scientist	Institute of Infection and Immunity, School of Medicine, Cardiff University
UK	Thomas	Hender	scientist	Institute of Infection and Immunity, School of Medicine, Cardiff University
UK	Patrick	Hogan	scientist	Institute of Infection and Immunity, School of Medicine, Cardiff University
UK	Habiba	Saif	scientist	Institute of Infection and Immunity, School of Medicine, Cardiff University
UK	Brekhna	Hassan	scientist	Institute of Infection and Immunity, School of Medicine, Cardiff University
UK	Ellis	Jones	scientist	Institute of Infection and Immunity, School of Medicine, Cardiff University
UK	Matthew	Barrell	scientist	Institute of Infection and Immunity, School of Medicine, Cardiff University
UK	Ian	Boostrom	scientist	Institute of Infection and Immunity, School of Medicine, Cardiff University
UK	Francis	Frayne	scientist	Institute of Infection and Immunity, School of Medicine, Cardiff University
UK	Jessica	Rees	scientist	Institute of Infection and Immunity, School of Medicine, Cardiff University
UK	Lim	Jones	clinician	Specialist Antimicrobial Chemotherapy Unit, Public Heath Wales, Cardiff, UK
UK	Susanna	Dunachie	scientist	Centre for Tropical Medicine and Global Health, Nuffield Department of Medicine, University of Oxford
UK	Brad	Spiller	scientist	Institute of Infection and Immunity, School of Medicine, Cardiff University
UK	Julian	Parkhill	scientist	Cambridge Infectious Diseases, Department of Veterinary Medicine, Cambridge University

Supplementary Table 5. BARNARDS group. List of countries enrolled in BARNARDS study, with individuals who provided help during the research, their forenames, surnames, position, and affiliation.

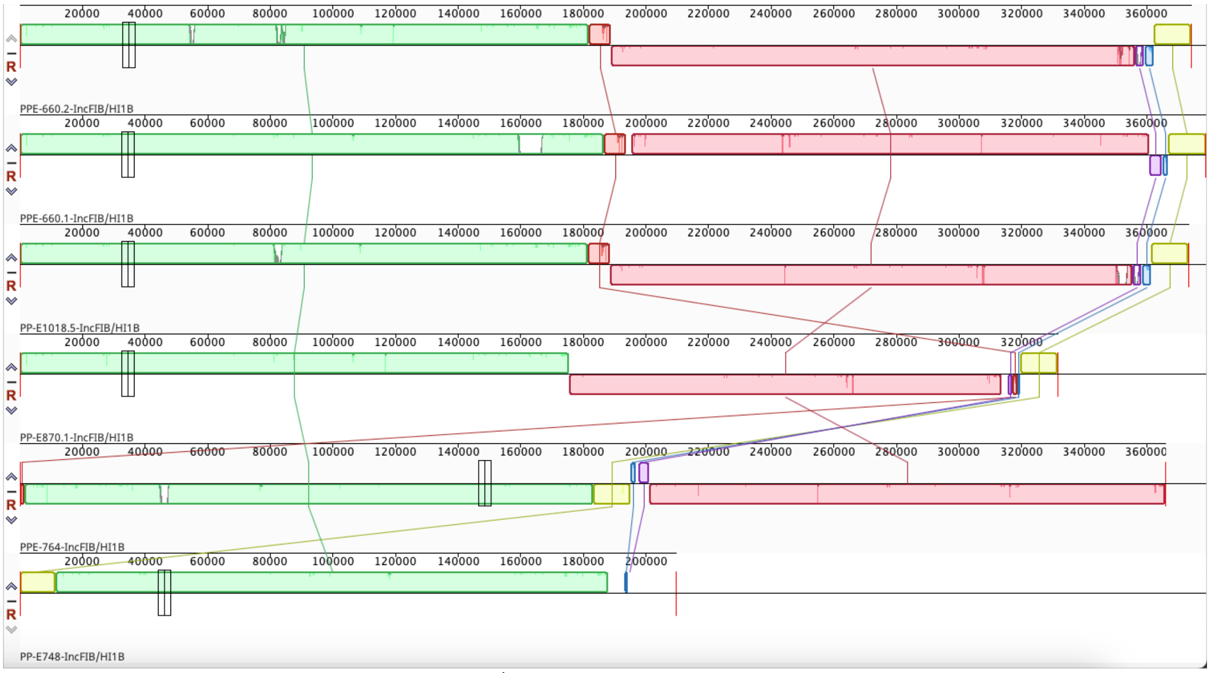

Supplementary Figure 1. A Mauve alignment[1] of six IncFIB/HI1B *bla*$_{\text{NDM-1}}$ plasmid sequences (including partial sequence pPP-E748; bottom) identified in multiple bacterial species sampled from surfaces in the hospital in Pakistan.Plasmids pPPE-660.1 and pPPE-660.2 were isolated from whole genome sequencing data from two bacterial species, a *K. michiganensis* and a *K. pneumoniae* from the same hospital surface sample. This is a particular example of possible plasmid transmission is between *K. pneumoniae, K. michiganensis* (sample PP-E660) and *Enterobacter roggenkampii* (pPPE-1018.5, third plasmid sequence in the series). Source data are provided as a Source Data file.

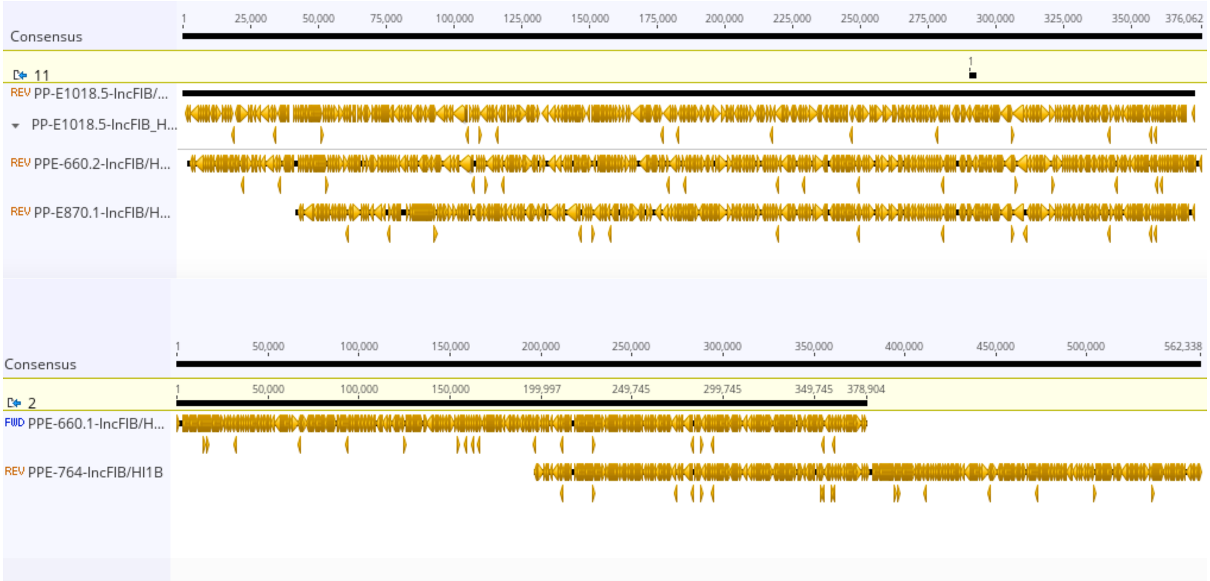

Supplementary Figure 2. A sequence alignment of five IncFIB/HI1B *bla*$_{\text{NDM-1}}$ plasmid sequences. Following a nucleotide alignment of the plasmids using MAFFT within Geneious, pPPE-660.1 and pPPE-660.2 were 70.2% homologous whereas pPP-E660.2 and pPPE-1018.5 were 98.5% homologous. Source data are provided as a Source Data file.

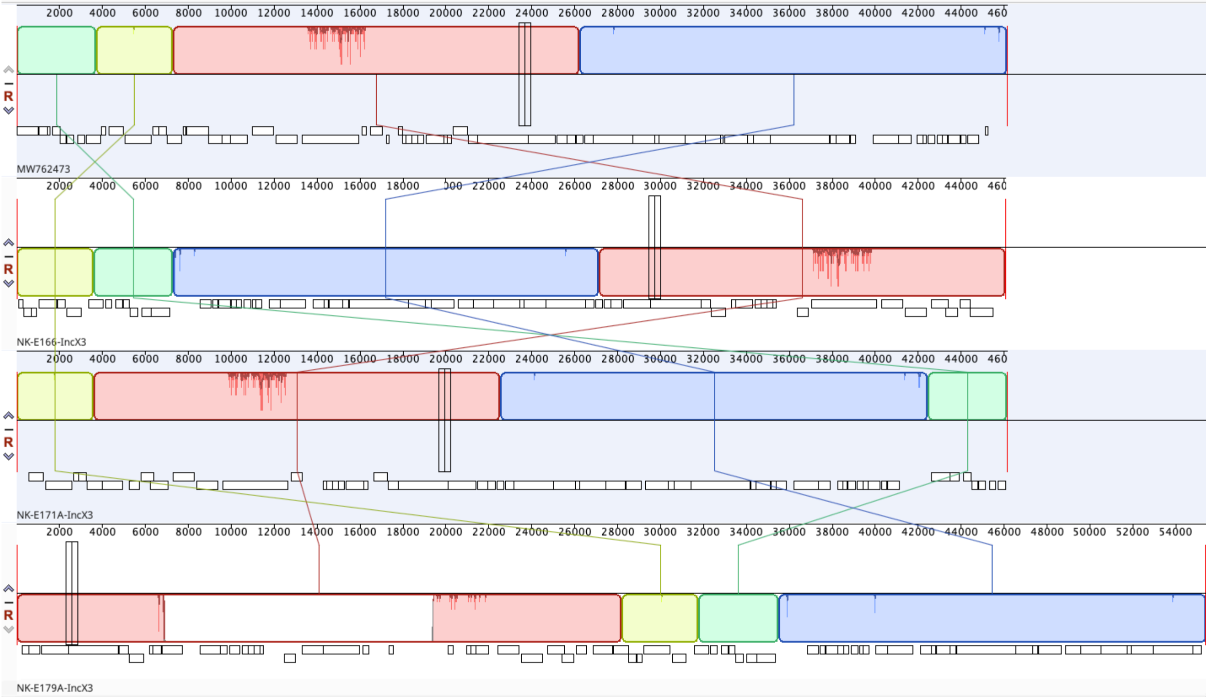

Supplementary Figure 3. A Mauve alignment[1] of IncX3 *bla*$_{\text{NDM-5}}$ plasmids from *Enterobacter hormaechei* isolates (singleton STs) cultured from hospital surfaces in Kano, Nigeria. Sequences were mapped to MW762473; a plasmid with *bla*$_{\text{NDM}}$ originating from an *E. coli* isolate, submitted to NCBI in 2021 in China. All plasmids originated from *E. hormaechei* isolates, one ST114, one ST696 and one ST947. All plasmids contained *bla*$_{\text{NDM-5}}$. All plasmids were marked as circular and complete from hybrid genome assembly using Unicycler with two plasmids pNK-E166-IncX3 and pNK-E171A-IncX3 being approximately 46kbp and one (pNK-E179A-IncX3) plasmid was larger at 55kbp. Source data are provided as a Source Data file.

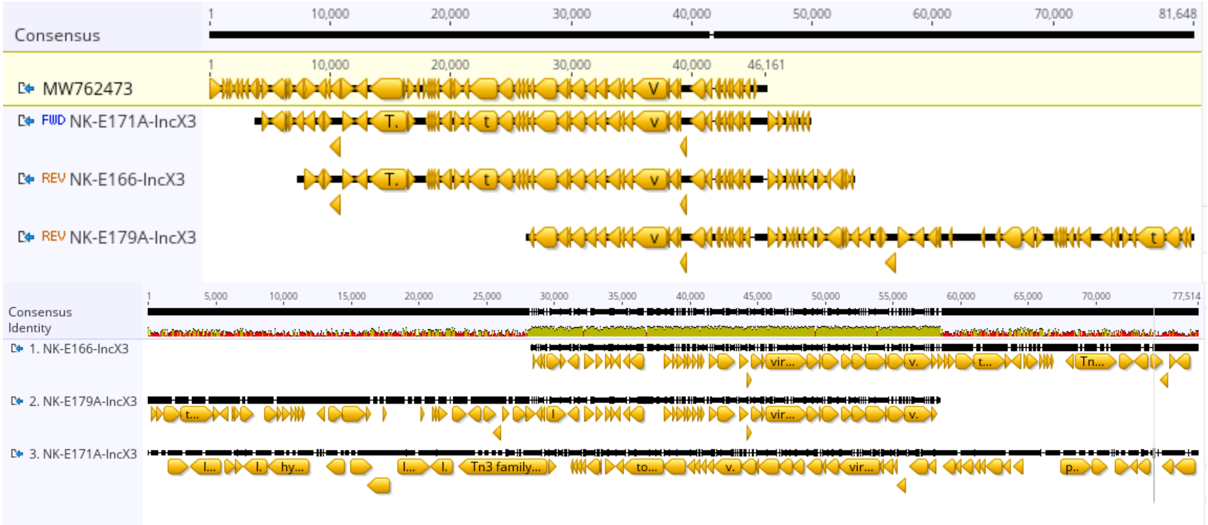

Supplementary Figure 4. A sequence alignment of *bla*$_{\text{NDM-5}}$ IncX3 plasmids from *Enterobacter hormaechei* isolates from Kano, Nigeria. Sequences were mapped to MW762473; a plasmid with *bla*$_{\text{NDM}}$ originating from an *E. coli* isolate, submitted to NCBI in 2021 in China. All plasmids originated from *E. hormaechei* isolates, one ST114, one ST696 and one ST947. All plasmids contained *bla*$_{\text{NDM-5}}$. All plasmids were marked as circular and complete from hybrid genome assembly using Unicycler with two plasmids pNK-E166-IncX3 and pNK-E171A-

IncX3 being approximately 46kbp and one (pNK-E179A-IncX3) plasmid was larger at 55kbp. Source data are provided as a Source Data file.

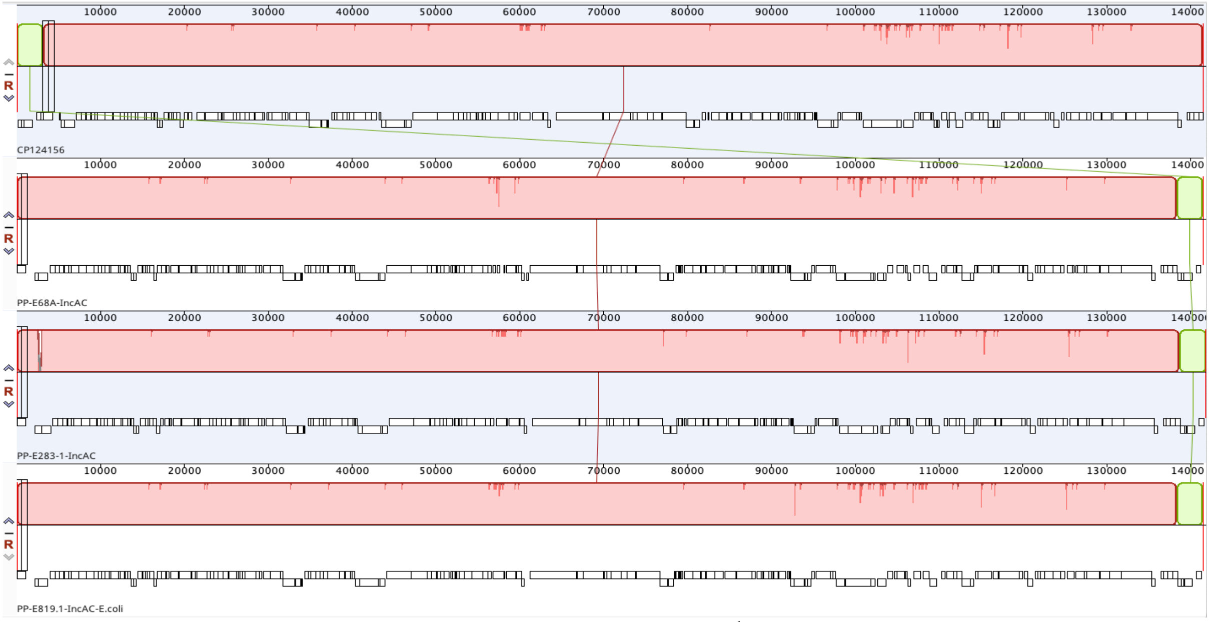

Supplementary Figure 5. This figure represents a mauve alignment[1] of IncAC plasmids with $bla_{\text{NDM-1}}$ including PP-E819.1, two plasmids from *Klebsiella* WGS data, and CP124156 which was identified as the most similar (99.99% pairwise identity; 141,528bp). CP124156 relates to an entry from USA in July 2023 and the plasmid originates in a *K. pneumoniae* strain. During analysis, we assembled 11 ColKP3-IncX3 plasmids (~50kb) with $bla_{\text{OXA-181}}$ and 11 larger *Inc*AC plasmids (~140kb) with $bla_{\text{NDM-1}}$ from the same 11 *K. pneumoniae* whole genome sequences. Moreover, an *Escherichia coli* isolate (PP-E819.1) cultured from an emergency neonatal care surface from Pakistan in 02/01/2017 carried both $bla_{\text{NDM-1}}$ and $bla_{\text{OXA-181}}$. Source data are provided as a Source Data file.

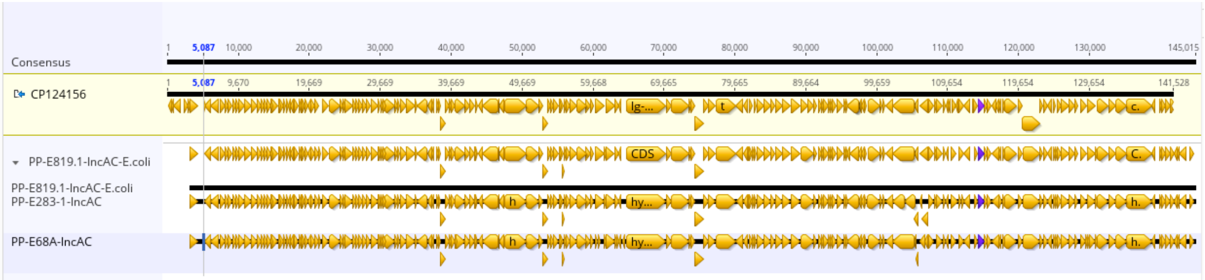

Supplementary Figure 6. Sequence alignment of 140kb IncAC plasmids carrying $bla_{\text{NDM-1}}$ evidences the similarity between the plasmid in *E. coli* pPP-E819-IncAC to pPP-E283-IncAC and pPP-E68A-IncAC found in *K. pneumoniae* isolates, indicating the IncAC plasmid carrying $bla_{\text{NDM-1}}$ (blue triangle) may already be widely disseminated. The mash dist metrics[2] between the plasmid from *E. coli* compared to the two plasmids (highest quality assembled and complete plasmids used as representatives from the clonal ST15 strain for comparison analysis) indicates between 982-992/1000 shared hashes. Source data are provided as a Source Data file.

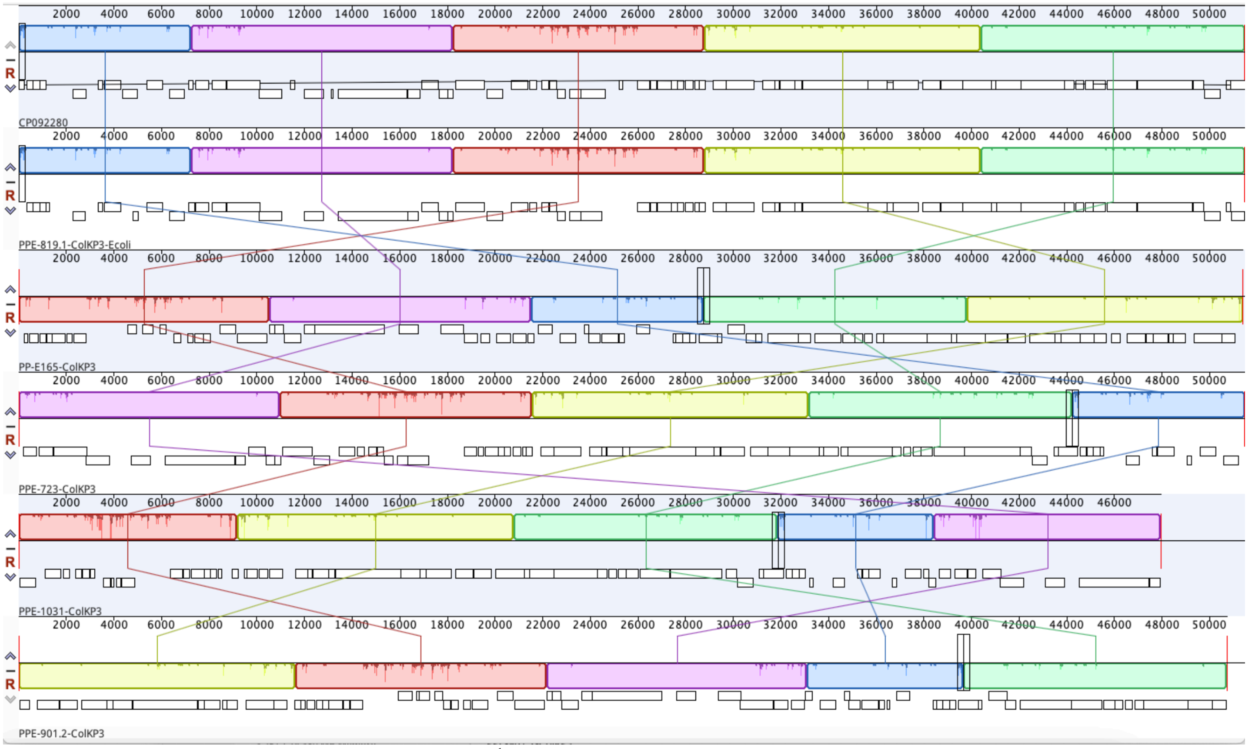

Supplementary Figure 7. A mauve alignment[1] of six plasmid sequences including CP092280, pPPE819.1-E. coli and four IncAC plasmids from ST15 K. pneumoniae isolates, indicating very similar IncAC plasmids. Analysis of ColKP3-IncX3 plasmids linked to the IncAC plasmids between ST15 K. pneumoniae and ST405 E. coli in Pakistan, the same single E. coli ST405 isolate carrying bla$_{NDM-1}$ on a 51,479bp IncAC plasmid. Following comparative analysis, CP092280 was identified as the closest genetic match (to pPPE-819.1) and was submitted to NCBI in 2023 from a group in China Liu, C et al (Unpublished). Following alignment of plasmid assemblies there was some genomic rearrangement within the ColKP3 plasmid assemblies, and varying levels of nucleotide mapping. Plasmids pPPE-819.1-ColKP3-Ecoli, pPPE-723-ColKP3, and pPPE-1031-ColKP3 share the greatest sequence homology when aligned against CP092280. pPPE-723-ColKP3 and pPPE-918.1-ColKP3 share 99.966% nucleotide bases, whereas pPPE-165-ColKP3 and pPPE-918.1-ColKP3 share 43.465% homology. Source data are provided as a Source Data file.

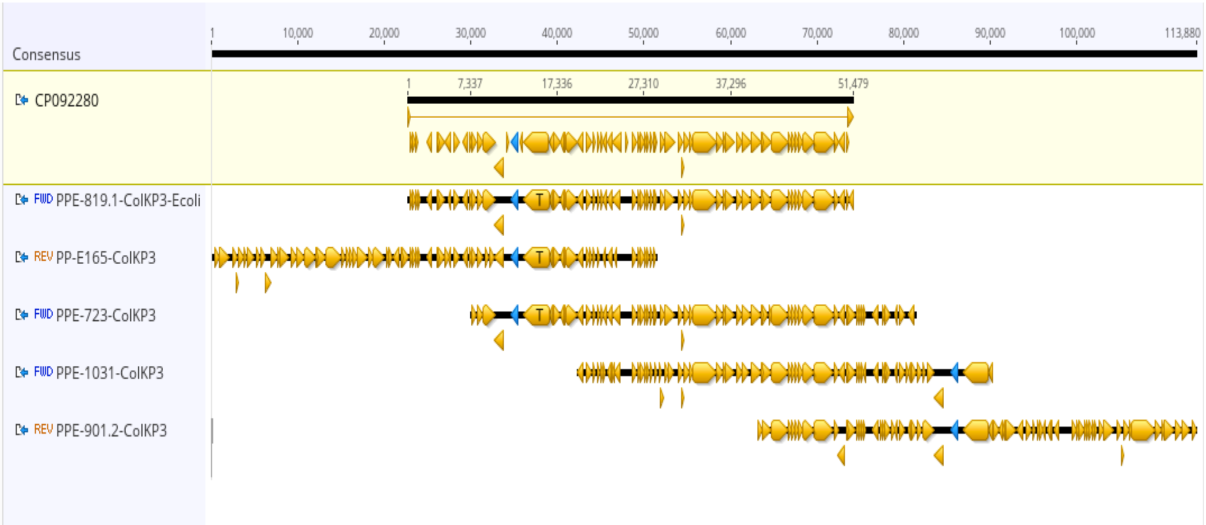

Supplementary Figure 8. A plasmid sequence alignment of five ColKP3-IncX3 plasmids including pPPE-819.1-E. coli. All sequences were aligned with CP092280 as the reference plasmid. Source data are provided as a Source Data file.

Supplementary references

1. Darling, A. C. E., Mau, B., Blattner, F. R. & Perna, N. T. Mauve: Multiple Alignment of Conserved Genomic Sequence With Rearrangements. *Genome Res.* **14 (7), 1394–1403**, (2004).
2. Ondov, B. D. *et al.* Mash: fast genome and metagenome distance estimation using MinHash. *Genome Biol.* **17 (1), 132**, (2016).
