# Supplementary material for: Colonisation of hospital surfaces from low- and middle-income countries by extended spectrum β-lactamase- and carbapenemase-producing bacteria
Source: Nat Commun. 2024 Mar 29;15:2758. doi: 10.1038/s41467-024-46684-z (PMC10980694; doi:10.1038/s41467-024-46684-z)
Supplement: Supplementary file 3 — Description of Additional Supplementary Files [file 41467_2024_46684_MOESM3_ESM.pdf]

<u>Description of Additional Supplementary Files</u>

Supplementary Data 1: Complete dataset.

Supplementary Data 2: Positive hospital surface swabs (HSS) for an antimicrobial resistance gene (ARG) out of the total 4,126 among the 309 surfaces collected.

Supplementary Data 3: Positive and negative hospital surface swabs (HSS) for an antimicrobial resistance gene (ARG) out of the total 4,126 among the six surface categories per each country and hospital site.

Supplementary Data 4: Number of isolates per hospital site carrying carbapenemase antimicrobial resistance genes (ARGs), according to PCR screening and bacterial species identification by MALDI-TOF MS.

Supplementary Data 5: Carbapenemase positive Gram-negative bacteria (GNB) isolates (128) out of the 175 total isolates recovered from hospital surface swabs (HSS), identified by MALDI-TOF MS and WGS.

Supplementary Data 6: Sequence types (ST) for the most commonly WGS identified species with a recognised multilocus sequence typing (MLST) scheme.

Supplementary Data 7: Classification of 309 different type of surfaces collected into six different surface categories.

Supplementary Data 8: List of genomes, submitted to NCBI (BioProject number PRJNA971772).
