# Supplementary material for: Colonisation of hospital surfaces from low- and middle-income countries by extended spectrum β-lactamase- and carbapenemase-producing bacteria
Source: Nat Commun. 2024 Mar 29;15:2758. doi: 10.1038/s41467-024-46684-z (PMC10980694; doi:10.1038/s41467-024-46684-z)
Supplement: Supplementary file 12 — Reporting Summary [file 41467_2024_46684_MOESM12_ESM.pdf]

nature portfolio

Corresponding author(s): Maria Nieto-Rosado

Last updated by author(s): Feb 8, 2024

Reporting Summary

Nature Portfolio wishes to improve the reproducibility of the work that we publish. This form provides structure for consistency and transparency in reporting. For further information on Nature Portfolio policies, see our Editorial Policies and the Editorial Policy Checklist.

Statistics

For all statistical analyses, confirm that the following items are present in the figure legend, table legend, main text, or Methods section.

n/a	Confirmed	
☐	☒	The exact sample size (*n*) for each experimental group/condition, given as a discrete number and unit of measurement
☐	☒	A statement on whether measurements were taken from distinct samples or whether the same sample was measured repeatedly
☒	☐	The statistical test(s) used AND whether they are one- or two-sided<br>*Only common tests should be described solely by name; describe more complex techniques in the Methods section.*
☒	☐	A description of all covariates tested
☒	☐	A description of any assumptions or corrections, such as tests of normality and adjustment for multiple comparisons
☒	☐	A full description of the statistical parameters including central tendency (e.g. means) or other basic estimates (e.g. regression coefficient) AND variation (e.g. standard deviation) or associated estimates of uncertainty (e.g. confidence intervals)
☐	☒	For null hypothesis testing, the test statistic (e.g. *F*, *t*, *r*) with confidence intervals, effect sizes, degrees of freedom and *P* value noted<br>*Give P values as exact values whenever suitable.*
☒	☐	For Bayesian analysis, information on the choice of priors and Markov chain Monte Carlo settings
☒	☐	For hierarchical and complex designs, identification of the appropriate level for tests and full reporting of outcomes
☒	☐	Estimates of effect sizes (e.g. Cohen's *d*, Pearson's *r*), indicating how they were calculated

Our web collection on statistics for biologists contains articles on many of the points above.

Software and code

Policy information about availability of computer code

Data collection	No software was used in the data collection. At the countries enrolled, surface information and location within the hospital, and date collection were reported in a document and sent to us. Regarding sampling procedure, HSS were collected based on enrollment at the neonatal wards but also when a sepsis case was reported, varying across countries and hospitals. Moreover, collection was limited by the feasibility of collecting samples in neonatal intensive care units. Additional data might be provided out of interest.
Data analysis	Guppy v5.0.11 and NVIDIA V100 GPUs<br>Unicycler (v0.4.9)<br>quast (v.5.2.0)<br>Pathogenwatch (v.3.13.10; https://pathogen.watch)<br>ABRicate (v0.9.7)<br>BIGSbd (v1.25.1)<br>Prokka (v1.12 and v1.14.5)<br>snippy (v4.6.0)<br>Snp-dists (v0.8.2)<br>Trimgalore (v0.6.4)<br>fastqc (v0.11.2)<br>Geneious (v2023.2.1)<br>mash dist (v2.2)<br>Adobe Illustrator v26.5<br>RStudio v4.3.0 (2023-04-21)—“Already Tomorrow”)<br>IBM SPSS Statistics (v25.0.0.1)(190)

For manuscripts utilizing custom algorithms or software that are central to the research but not yet described in published literature, software must be made available to editors and reviewers. We strongly encourage code deposition in a community repository (e.g. GitHub). See the Nature Portfolio guidelines for submitting code & software for further information.

Data

Policy information about availability of data

All manuscripts must include a data availability statement. This statement should provide the following information, where applicable:

- Accession codes, unique identifiers, or web links for publicly available datasets
- A description of any restrictions on data availability
- For clinical datasets or third party data, please ensure that the statement adheres to our policy

Databases used for in silico analysis in this work are PlasmidFinder, CARD, Enterobase, and PubMLST. Coloured maps in the paper were created using MapChart (https://www.mapchart.net).
The dataset generated in this study has been deposited in the Figshare repository (https://doi.org/10.6084/m9.figshare.23790360). All raw data is available within this manuscript as Supplementary Data files. Genomes are available in the NCBI database under BioProject number PRJNA971772 (and accession codes/accessible links are provided in Supplementary Data file 9). The plasmid analysis data generated in this study for evidence of transmission is available in the Supplementary Information file.

Research involving human participants, their data, or biological material

Policy information about studies with human participants or human data. See also policy information about sex, gender (identity/presentation), and sexual orientation and race, ethnicity and racism.

Reporting on sex and gender	na
Reporting on race, ethnicity, or other socially relevant groupings	na
Population characteristics	na
Recruitment	na
Ethics oversight	na

Note that full information on the approval of the study protocol must also be provided in the manuscript.

Field-specific reporting

Please select the one below that is the best fit for your research. If you are not sure, read the appropriate sections before making your selection.

☒ Life sciences ☐ Behavioural & social sciences ☐ Ecological, evolutionary & environmental sciences

For a reference copy of the document with all sections, see nature.com/documents/nr-reporting-summary-flat.pdf

Life sciences study design

All studies must disclose on these points even when the disclosure is negative.

Sample size

6,290 hospital surface swabs were processed from 10 hospital sites (BC, BK, ES, NK, NN, NW, PP, RK, RU, ZAT) in six low and middle-income countries. n=1,024 were collected from Bangladesh (BC n=410, BK n=614), n=253 from Ethiopia (ES), n=1,566 from Nigeria (NK n=472, NN=695, NW=399), n=1,033 from Pakistan (PP), n=927 from Rwanda (RK n=467, RU n=460), and n=1,487 from South Africa (ZAT).

No statistical methods were employed to predetermine sample size. Hospital surface swabs were collected based on enrollment at the neonatal wards, but also when a sepsis case was reported. Moreover, the feasibility of collecting samples in neonatal intensive care units was limited, therefore type of surface collected depended on workload and ward accessibility at the sampling moment, and not per strict requisition. Therefore, we did not request for a specific sample size per hospital or per ward.

Data exclusions

No. However, to study the correlation between presence of ARG and surface type, n=4,126/6,290 samples were included, as they contained appropriate metadata following data cleaning. To study the correlation between presence of ARG and timeline, n=4,662/6,290 samples contained appropriate metadata following data cleaning.

Replication

No. Strict replication was not performed as part of the study design, and only one swab was requested for collection per type of surface and per month, as we aimed for an aggregated analysis to look at the antimicrobial resistance genes producing-Gram-negative bacteria carriage by neonates with sepsis and their mothers. However, same surface type was intended to be sampled throughout the study timeline. It was likely that type of surfaces considered high touch were sampled more than once throughout the sampling period, however this data was not

	recorded as replicates.
Randomization	No. Randomisation is not relevant. We processed all hospital surface swabs collected and received. However, HSS were collected based on enrollment at the neonatal wards, but also when a sepsis case was reported. Moreover, the feasibility of collecting samples in neonatal intensive care units was limited, therefore type of surface collected depended on workload and ward accessibility at the sampling moment, and not per strict requisition.
Blinding	No. Blinding is not relevant in this study. However, a variety of samples were collected within the ward each month on regular visits to the ward. Cleaning staff was not warned, so as to avoid interfering in usual cleaning practises.

Reporting for specific materials, systems and methods

We require information from authors about some types of materials, experimental systems and methods used in many studies. Here, indicate whether each material, system or method listed is relevant to your study. If you are not sure if a list item applies to your research, read the appropriate section before selecting a response.

Materials & experimental systems

n/a	Involved in the study
☒	☐ Antibodies
☒	☐ Eukaryotic cell lines
☒	☐ Palaeontology and archaeology
☒	☐ Animals and other organisms
☒	☐ Clinical data
☒	☐ Dual use research of concern
☒	☐ Plants

Methods

n/a	Involved in the study
☒	☐ ChIP-seq
☒	☐ Flow cytometry
☒	☐ MRI-based neuroimaging
